# GP consultation rates for sequelae after acute covid-19 in patients managed in the community or hospital in the UK: population based study

**DOI:** 10.1136/bmj-2021-065834

**Published:** 2021-12-29

**Authors:** Hannah R Whittaker, Claudia Gulea, Ardita Koteci, Constantinos Kallis, Ann D Morgan, Chukwuma Iwundu, Mark Weeks, Rikisha Gupta, Jennifer K Quint

**Affiliations:** 1 National Heart and Lung Institute, Imperial College London, London, UK; 2 NIHR Imperial Biomedical Research Centre, London, UK

## Abstract

**Objectives:**

To describe the rates for consulting a general practitioner (GP) for sequelae after acute covid-19 in patients admitted to hospital with covid-19 and those managed in the community, and to determine how the rates change over time for patients in the community and after vaccination for covid-19.

**Design:**

Population based study.

**Setting:**

1392 general practices in England contributing to the Clinical Practice Research Datalink Aurum database.

**Participants:**

456 002 patients with a diagnosis of covid-19 between 1 August 2020 and 14 February 2021 (44.7% men; median age 61 years), admitted to hospital within two weeks of diagnosis or managed in the community, and followed-up for a maximum of 9.2 months. A negative control group included individuals without covid-19 (n=38 511) and patients with influenza before the pandemic (n=21 803).

**Main outcome measures:**

Comparison of rates for consulting a GP for new symptoms, diseases, prescriptions, and healthcare use in individuals admitted to hospital and those managed in the community, separately, before and after covid-19 infection, using Cox regression and negative binomial regression for healthcare use. The analysis was repeated for the negative control and influenza cohorts. In individuals in the community, outcomes were also described over time after a diagnosis of covid-19, and compared before and after vaccination for individuals who were symptomatic after covid-19 infection, using negative binomial regression.

**Results:**

Relative to the negative control and influenza cohorts, patients in the community (n=437 943) had significantly higher GP consultation rates for multiple sequelae, and the most common were loss of smell or taste, or both (adjusted hazard ratio 5.28, 95% confidence interval 3.89 to 7.17, P<0.001); venous thromboembolism (3.35, 2.87 to 3.91, P<0.001); lung fibrosis (2.41, 1.37 to 4.25, P=0.002), and muscle pain (1.89, 1.63 to 2.20, P<0.001); and also for healthcare use after a diagnosis of covid-19 compared with 12 months before infection. For absolute proportions, the most common outcomes ≥4 weeks after a covid-19 diagnosis in patients in the community were joint pain (2.5%), anxiety (1.2%), and prescriptions for non-steroidal anti-inflammatory drugs (1.2%). Patients admitted to hospital (n=18 059) also had significantly higher GP consultation rates for multiple sequelae, most commonly for venous thromboembolism (16.21, 11.28 to 23.31, P<0.001), nausea (4.64, 2.24 to 9.21, P<0.001), prescriptions for paracetamol (3.68, 2.86 to 4.74, P<0.001), renal failure (3.42, 2.67 to 4.38, P<0.001), and healthcare use after a covid-19 diagnosis compared with 12 months before infection. For absolute proportions, the most common outcomes ≥4 weeks after a covid-19 diagnosis in patients admitted to hospital were venous thromboembolism (3.5%), joint pain (2.7%), and breathlessness (2.8%). In patients in the community, anxiety and depression, abdominal pain, diarrhoea, general pain, nausea, chest tightness, and tinnitus persisted throughout follow-up. GP consultation rates were reduced for all symptoms, prescriptions, and healthcare use, except for neuropathic pain, cognitive impairment, strong opiates, and paracetamol use in patients in the community after the first vaccination dose for covid-19 relative to before vaccination. GP consultation rates were also reduced for ischaemic heart disease, asthma, and gastro-oesophageal disease.

**Conclusions:**

GP consultation rates for sequelae after acute covid-19 infection differed between patients with covid-19 who were admitted to hospital and those managed in the community. For individuals in the community, rates of some sequelae decreased over time but those for others, such as anxiety and depression, persisted. Rates of some outcomes decreased after vaccination in this group.

## Introduction

The covid-19 pandemic continues to challenge global public health, not least because of the growing realisation that the effects of covid-19 can affect individuals beyond the period of acute presentation. Along with persistent symptoms, evidence is also emerging of new end organ dysfunction in those who recover from acute infection, with potentially negative effects on cardiovascular,^1^ respiratory,^2^ metabolic,^3^ haematological,^4^ psychological,^5^ and neurological health.^6^

Although working definitions of long covid have been established,^7^ ^8^ understanding of the short and long term health consequences after covid-19 infection is limited. One of the main factors precluding a comprehensive understanding of the sequelae after acute covid-19 infection is the focus in the published literature and ongoing longitudinal studies on assessing outcomes in patients admitted to hosptial.^9^ But about 80% of patients with covid-19 have mild disease, with only 3.5% of patients in England requiring admission to hospital at the start of the first wave,^10^ and 6.4% requiring admission to hospital in total since the start of the pandemic.^11^ ^35^ Also, evidence is emerging of a considerable burden of sequelae after acute covid-19 infection in patients in the community (that is, patients not requiring admission to hospital).^12^ ^13^ Notably, patient group letters,^14^ surveys,^15^ and qualitative studies have highlighted a high proportion of patients with persistent debilitating symptoms.^16^ Current estimates of the prevalence of long covid are highly variable but many are limited in their generalisability because of small cohort sizes and selection biases. Few studies, however, have compared outcomes across the range of severities of covid-19.^17^ ^18^ Understanding the nature and burden of sequelae after acute covid-19 across different patient groups is crucial for effective rehabilitation services that can provide adequate and tailored support to those affected.

In this study, we used a large UK primary care longitudinal dataset, broadly representative of the English population, to investigate new symptoms, diseases, prescriptions, and healthcare use, recorded in primary care, for two separate patient cohorts after acute covid-19 infection: patients admitted to hospital and patients managed in the community. Also, we used two comparison cohorts to contextualise the findings: a negative control group of people without covid-19, to understand healthcare use during the pandemic; and an historical cohort of patients with influenza before the pandemic, to understand whether associations in our covid-19 cohort were caused by acute respiratory infection or specifically by covid-19. Focusing on the large population of patients with covid-19 in the community and taking advantage of the length of follow-up now available, we explored how this burden changed over time. Considering the emerging evidence of a possible improvement in symptoms after vaccination for covid-19,^19^ we also compared the prevalence of sequalae after covid-19 before and after vaccination in patients in the community who had sequelae after acute covid-19 infection.

## Methods

### Data source

We used the Clinical Practice Research Datalink (CPRD) Aurum, a nationally representative database of anonymised primary care electronic healthcare records, which holds data on symptoms, diagnoses, prescriptions, test results, immunisations, consultations, admissions to hospital, and specialist referrals for more than 13 million patients in England, covering about 23% of the English population.^20^ As one of the largest longitudinal databases worldwide, its use has been extensively validated.^21^ Clinical information is entered with SNOMED CT (systematised nomenclature of medicine-clinical terms) codes, and prescriptions are recorded with British National Formulary codes. Data from patient records are used only if they meet a certain standard of quality. For participating general practices, data from secondary care services are also fed back into primary care records and the CPRD Aurum.

### Study populations

#### Two covid-19 cohorts: those admitted to hospital and those managed in the community

**Fig 1:**
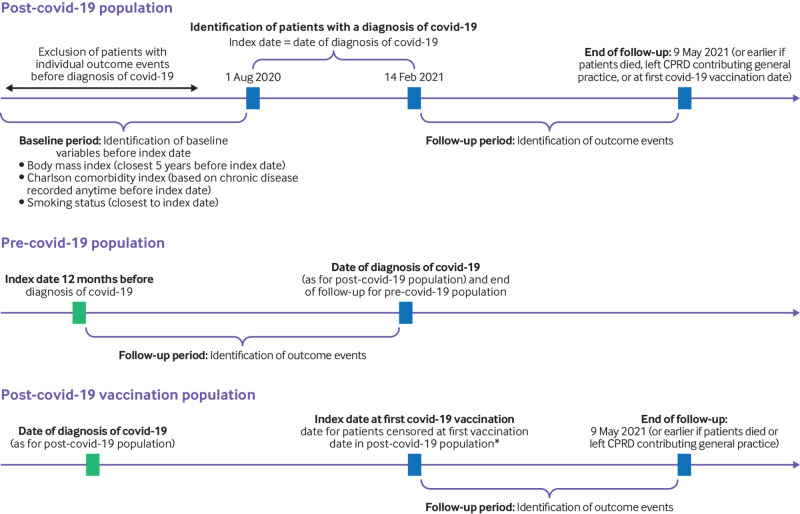
Study design. Follow-up period for the pre-covid-19 population (patients before having a covid-19 diagnosis) was defined in the same way as for the post-covid-19 population (patients after having a covid-19 diagnosis). The post-covid-19 vaccination population was defined as all patients who tested positive for covid-19, as defined for the post-covid population, and received a first vaccine dose for covid-19 at a later date. Start of follow-up for the vaccination population was the date of vaccination and end of follow-up was the same as for the post-covid population. CPRD=Clinical Practice Research Datalink Aurum. *In patients with at least one symptom outcome post-covid-19

The study population included individuals aged ≥18 years with a positive test for covid-19 and registered with a general practice contributing to the CPRD Aurum. Patients with covid-19 were identified from 1 August 2020 to 14 February 2021. The patient’s index date was the date of the diagnosis of covid-19. Eligible patients were categorised as managed in the community or admitted to hospital, depending on hospital admission for covid-19 within two weeks of their index date. Patients were censored at the earliest date of transfer out of practice, date of first covid-19 vaccination, death, or last collection date (9 May 2021) (fig 1). Patients with evidence of any of the investigated outcomes before a diagnosis of covid-19 were excluded from individual analyses so that we captured outcomes caused by covid-19 infection rather than pre-existing factors. Outcomes were also determined 12 months before each patient’s index date to evaluate outcome patterns before and after covid-19 infection in the same patients.

Baseline characteristics included the most recent measurement of body mass index within five years of the index date. We chose a period of five years because of high levels of data missingness for body mass index (page 3, supplementary material). Smoking status and the Charlson comorbidity index were identified at any time point before a diagnosis of covid-19, and we extracted data closest to the start of follow-up (fig 1). The Charlson comorbidity index was identified with a previously published algorithmm.^22^

#### Negative covid-19 control cohort

The covid-19 pandemic has substantially changed the epidemiology of disease because of changes in healthcare provision, social distancing, and healthcare seeking behaviours. Therefore, to help contextualise our findings and determine whether outcomes after covid-19 were caused by the acute infection or confounded by other temporal aspects of the pandemic, we also studied patients who had been seen by their general practitioner (GP) for suspected covid-19 or because of contact with an individual with covid-19 but who never had a positive covid-19 test. The index date was when patients saw their GP for suspected covid-19 between 1 August 2020 and 14 February 2021. Patients with a diagnosis of covid-19 before or after these dates or who were admitted to hospital within two weeks of the index date were excluded. Patients were censored at the earliest date of the date they transferred out of practice, date of first covid-19 vaccination, death, or last collection date (9 May 2021). Similar to the main covid-19 cohort, outcomes were also determined 12 months before each patient’s index date, and patients with evidence of any of the investigated outcomes before their index date were excluded from individual analyses.

#### Influenza cohort

To contextualise our main findings, we included a population of patients with a diagnosis of influenza who were not admitted to hospital. The index date was the date of the diagnosis of influenza recorded before the pandemic, from 1 August 2018 to 14 February 2019. Patients having a diagnosis of influenza in the year before or who were admitted to hospital within two weeks of the index date were excluded. Patients were censored at the earliest date of transfer out of practice, death, or last collection date (9 May 2019). Similar to the analyses in the main covid-19 and negative covid-19 cohorts, GP consultation rates before and after a diagnosis of influenza were compared in patients with no evidence of any of the investigated outcomes before their index date.

#### Post-vaccine cohort

To understand the association between vaccination against covid-19 and persistence of symptoms, diseases, drug prescriptions, and healthcare use in patients in the community who had sequelae after acute covid-19, we examined the occurrence of each outcome in a subgroup of patients who had received at least one dose of any of the three approved covid-19 vaccines in the UK (Pfizer-BioNTech, Moderna, or Oxford-AstraZeneca). For these patients, the start of follow-up was the date of their covid-19 vaccination, and the end of follow-up was the earliest of the last collection date or death.

### Definition of outcomes

Outcomes included symptoms and diseases most likely to affect patients after infection, guided by the previous literature. We also investigated prescriptions for selected drugs and levels of healthcare use. Code lists were reviewed by a clinician and can be accessed at www://github.com/NHLI-Respiratory-Epi/Long_covid_codelists.

Symptoms were chosen according to the National Institute for Health and Care Excellence (NICE) 2020 guideline on common symptoms persisting after acute infection,^7^ and included breathlessness, cough, chest tightness, chest pain, palpitations, abdominal pain, anorexia or reduced appetite, nausea, diarrhoea, joint and muscle pain, skin rashes, headache, dizziness, insomnia, cognitive impairment, delirium, paraesthesia, tinnitus, earache, sore throat, loss of smell or taste, or both, and fatigue, fever, and pain.

We looked at non-communicable diseases, including hypertension, ischaemic heart disease, heart failure, peripheral arterial disease, stroke or transient ischaemic attack, or both, asthma, pulmonary fibrosis, inflammatory bowel disease, liver disease, gastro-oesophageal reflux disease, diabetes, thyroid and adrenal disorders, renal failure, arthritis, venous thromboembolism, anaemia, delirium, anxiety, and depression.

We investigated drug prescriptions, including bronchodilators, inhaled corticosteroids, paracetamol, non-steroidal anti-inflammatory drugs, opiates, neuropathic pain drugs, and diuretics. New healthcare use included number of visits to the GP or accident and emergency department, hospital admissions, and outpatient appointments.

For the outcomes, symptoms, prescriptions, and healthcare use, we defined a new event as the first occurrence four weeks after a diagnosis of covid-19, in line with the current NICE definition of long covid. For disease outcomes, we defined a new event as the first occurrence after a diagnosis of covid-19. Patients with any of the defined symptoms in the preceding month before a diagnosis of covid-19 were excluded from the analyses. For diseases and prescriptions, we applied a 12 month exclusion window (fig 1). We used a similar approach when we assessed outcomes 12 months before a diagnosis of covid-19.

To investigate the temporal pattern of the occurrence of each outcome after covid-19 in patients in the community, we described the occurrence of outcomes within each month of follow-up, from four weeks after a diagnosis of covid-19 until the end of the follow-up period.

### Statistical analysis

#### Main analysis

Baseline characteristics are presented as frequencies (percentages) for categorical data and median (interquartile range) for continuous variables. Cox regression models with cluster correlated robust sandwich variance estimators,^23^ to take into account clustering of observation time periods within patients (and time since the index date), were used to estimate hazard ratios and 95% confidence intervals to compare GP consultation rates for each outcome (symptom, disease, and drug prescription) between two time points (after covid-19 infection and 12 months before a diagnosis of covid-19), separately for the two covid-19 cohorts (patients in the community and those admitted to hospital). We also performed this analysis in the negative control and influenza cohorts. Hazard ratios were adjusted for age, sex, Charlson comorbidity index, body mass index, and smoking status. Rates for healthcare use (with time since the index date) before and after covid-19 were compared with negative binomial regression models. To adjust for clustering of observation time periods within patients, we estimated incidence rate ratios (with 95% confidence intervals) based on cluster correlated robust sandwich variance estimators.^23^ To account for multiple testing, we used the Bonferroni correction to adjust the threshold for statistical significance.

#### Post-covid-19 monthly outcome rates

We calculated GP consultation rates for symptoms, diseases, and prescriptions for each month of follow-up for patients in the community by dividing the total number of events (outcomes) by person time in each one month time interval.

#### Post-vaccination analysis

We used negative binomial regression models to assess the association between receiving the covid-19 vaccine and rate of each outcome after covid-19 in patients in the community with ongoing symptoms after the acute covid-19 infection. Estimated incidence rate ratios and 95% confidence intervals were calculated to compare GP consultation rates between the date of a covid-19 diagnosis and the date of vaccination with those rates between the date of vaccination and 9 May 2021, date of death, or date the patient left the practice. Incidence rate ratios and 95% confidence intervals were adjusted for time since a diagnosis of covid-19 and, where data allowed, other confounders (that is, age, sex, Charlson comorbidity index, body mass index, and smoking status).

#### Sensitivity analyses

We extended the two week window for identifying patients with covid-19 admitted to hospital to three and four week windows. In a further analysis, we identified symptom and prescription outcomes two weeks after a diagnosis of covid-19 instead of four weeks. We also identified body mass index within one and two years of the index date. We repeated the Cox regression analyses in the covid-19 cohort who were managed in the community, without excluding patients with previous events. Lastly, outcome events could decline over time and therefore we compared outcomes before and after vaccination by comparing the rate of outcome events in the month before vaccination with the rate after vaccination. All statistical analyses were conducted with Stata 17. Graphs were created with R version 4.0.3.

### Patient and public involvement

Although no specific patient or user groups were involved in the design of this study, during revision of the manuscript, we took the opportunity to discuss the paper with patients who were attending respiratory outpatient clinics over the course of a few weeks and who themselves had either had covid-19 or were aware of someone who had. Patients were supportive of and could see the need for the study. Ongoing patient involvement will be facilitated by communication of our findings to patients in long covid clinics to guide their ongoing management and care.

## Results

Between August 2020 and February 2021, 456 002 patients received a covid-19 diagnosis in the cohort, of whom 18 059 were admitted to hospital and 437 943 were managed in the community. Table 1 summarises the baseline characteristics. The geographical distribution of patients in the cohort spanned across England, with the largest patient numbers from London and the south east (31.9%), the north west (22.1%), and the West Midlands (16.6%) (table S1).

**Table 1:** Baseline characteristics for patients with covid-19 managed in the community, those admitted to hospital with covid-19, and those with an influenza diagnosis. Data are number (%) of patients

Baseline characteristics	Community covid-19	Admitted to hospital with covid-19	Influenza cohort
**Sex**			
Male	194 583 (44.4)	9140 (50.6)	8699 (39.9)
Female	243 360 (55.6)	8919 (49.4)	13 104 (60.1)
**Age**			
18-30	115 951 (26.5)	1048 (5.8)	3434 (15.8)
31-40	91 735 (21.0)	1735 (9.6)	4388 (20.1)
41-50	85 517 (19.5)	2398 (13.3)	4507 (20.7)
51-60	80 817 (18.5)	3642 (20.2)	4394 (20.2)
61-70	37 502 (8.6)	3127 (17.3)	2638 (12.1)
71-80	14 860 (3.4)	2972 (16.5)	1627 (7.5)
>80	11 561 (2.6)	3137 (17.4)	815 (3.7)
**Body mass index**			
≤18.5	6426 (1.5)	307 (1.7)	362 (1.7)
18.5-24.5	92 634 (21.2)	2897 (16.0)	5201 (23.9)
25.0-29.9	95 407 (21.8)	4551 (25.2)	5237 (24.0)
≥30.0	94 168 (21.5)	6854 (38.0)	5078 (23.3)
Unknown	149 308 (34.1)	3450 (19.0)	5925 (27.2)
**Smoking status**			
Current smoker	74 294 (17.1)	4054 (22.5)	4961 (22.8)
Ex-smoker	204 342 (46.7)	11 459 (63.5)	10 389 (47.7)
Never smoked	141 500 (32.3)	2390 (13.2)	6212 (28.5)
Unknown	17 294 (4.0)	156 (0.9)	241 (1.1)
**Baseline diseases (CCI)**			
0	263 681 (60.2)	5484 (30.4)	11 627 (53.3)
1-2	134 781 (30.8)	6348 (35.2)	7449 (34.2)
3-4	28 204 (6.4)	3621 (20.1)	1897 (8.7)
5-6	8516 (1.9)	1649 (9.1)	613 (2.8)
>6	2761 (0.6)	957 (5.3)	217 (1.0)
**Total No of patients**	437 943	18 059	21 803

CCI= Charlson comorbidity index.

Patients admitted to hospital with covid-19 were older (median age 61, interquartile range 48-76 *v* 43, 30-55), were more likely to be men (50.6% *v* 44.4%), overweight or obese (63.2% *v* 43.3%), and ex-smokers or current smokers (86.0% *v* 63.8%) than those managed in the community. Although patients admitted to hospital were more likely to have comorbidities, the median number of comorbidities was low in both groups. Of those people who had a covid-19 diagnosis and were managed in the community, 0.95% died during follow-up versus 9.7% of those admitted to hospital.

### Post-covid-19 versus pre-covid-19 outcomes in patients admitted to hospital

**Fig 2:**
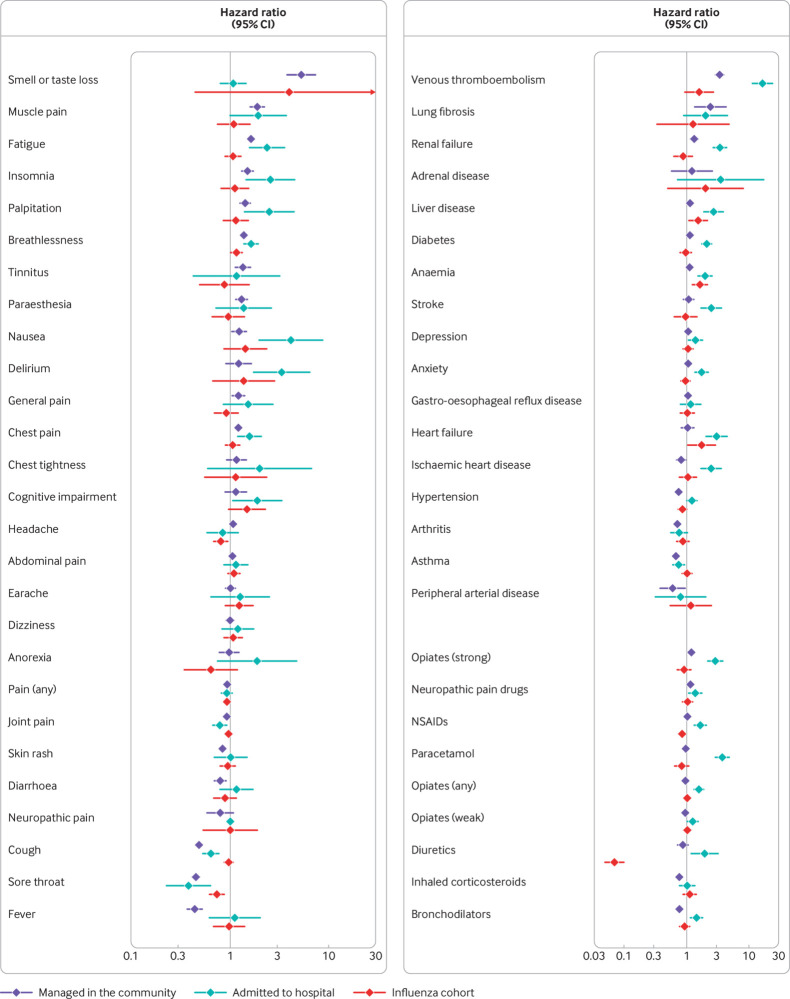
General practitioner consultation rates for clinical outcomes after covid-19 infection compared with 12 months before covid-19 infection, in patients admitted to hospital with covid-19, in patients managed in the community, and in the influenza cohort. Forest plots show hazard ratios (95% confidence interval) for each outcome developed versus each outcome not developed, separately, during follow-up. Analyses were adjusted for age, sex, body mass index, Charlson comorbidity index, and smoking status. NSAIDs=non-steroidal anti-inflammatory drugs

Median follow-up time was 2.2 months (interquartile range 1.3-3.5). The most common symptoms ≥4 weeks after a diagnosis of covid-19 in patients admitted to hospital were joint pain (2.7% of patients), breathlessness (2.8%), and cough (1.4%). Patients admitted to hospital had the highest GP consultation rates after acute covid-19 for nausea (adjusted hazard ratio 4.64, 95% confidence interval 2.34 to 9.21, P<0.001), followed by delirium (3.24, 1.77 to 5.94, P<0.001), palpitations (2.55, 1.61 to 4.05, P<0.001), fatigue (2.52, 1.81 to 3.51, P<0.001), and insomnia (2.17, 1.36 to 3.49, P=0.001) relative to 12 months before infection. The GP consultation rates for breathlessness were also higher in patients admitted to hospitalised compared with 12 months previously (fig 2, and tables S2 and S6).

At least four weeks after a diagnosis of covid-19, 3.5% of patients admitted to hospital developed venous thromboembolism, 2.5% developed diabetes, and 1.8% received a hypertension diagnosis. Relative to the period 12 months before their covid-19 infection, patients admitted to hospital had significantly higher GP consultation rates for venous thromboembolism (adjusted hazard ratio 16.21, 95% confidence interval 11.28 to 23.31, P<0.001) after covid-19. The rate at which patients admitted to hospital consulted their GP for renal failure (3.42, 2.67 to 4.38, P<0.001), heart failure (3.02, 2.07 to 4.42, P<0.001), liver disease (2.71, 1.92 to 3.83, P<0.001), and stroke (2.49, 1.73 to 3.59, P<0.001) was also higher after covid-19, and to a lesser extent also for ischaemic heart disease, anaemia, diabetes, and anxiety (fig 2, and tables S3 and S6).

**Fig 3:**
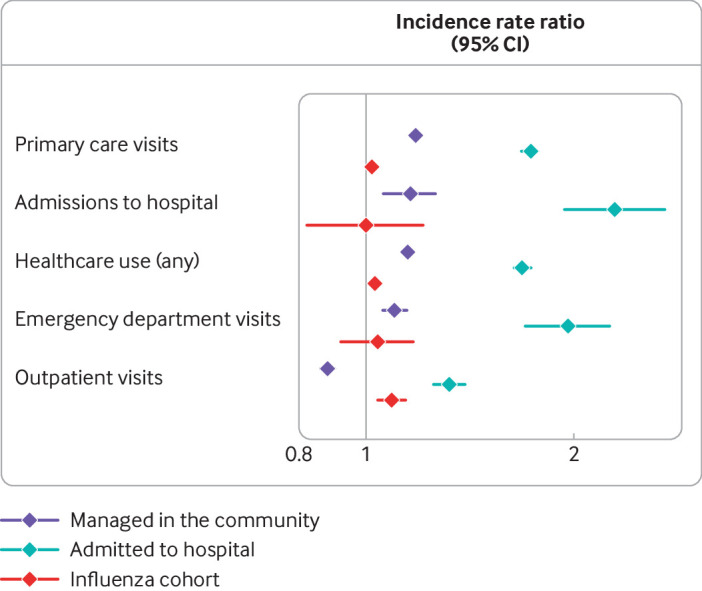
Incidence of healthcare use outcomes after covid-19 infection compared with 12 months before covid-19 infection, in patients admitted to hospital with covid-19, in patients managed in the community, and in the influenza cohort. Forest plots show incidence rate ratios (95% confidence interval) for each outcome developed during follow-up. Analyses were adjusted for age, sex, body mass index, Charlson comorbidity index, and smoking status

The most commonly prescribed new drugs ≥4 weeks after a diagnosis of covid-19 were opiates (2.2% of patients), paracetamol (1.8%), and non-steroidal anti-inflammatory drugs (1.6%). We found the greatest change in the level of prescriptions for paracetamol after covid-19 relative to 12 months before infection (adjusted hazard ratio 3.68, 95% confidence interval 2.86 to 4.74, P<0.001), followed by diuretics (1.93, 1.19 to 3.14, P=0.008), non-steroidal anti-inflammatory drugs (1.65, 1.34 to 2.04, P<0.001), and any opiate (1.57, 1.32 to 1.87, P<0.001), (fig 2, and tables S4 and S6). Patients admitted to hospital were also prescribed more bronchodilators, neuropathic pain drugs, and weak opiates. Lastly, in terms of healthcare use, patients admitted to hospital for covid-19 had more admissions to hospital (adjusted incidence rate ratio 2.29, 95% confidence interval 1.94 to 2.70, P<0.001), visits to the emergency department (1.96, 1.70 to 2.25, P<0.001), visits to primary care (1.73, 1.68 to 1.77, P<0.001), and outpatient appointments (1.32, 1.25 to 1.39, P<0.001) after covid-19 compared with 12 months before infection. For the overall increase in healthcare use, the incidence rate ratio was 1.68 (95% confidence interval 1.64 to 1.73, P<0.001) (fig 3, and tables S5 and S6).

### Post-covid-19 versus pre-covid-19 outcomes in individuals managed in the community

Median follow-up time was 3.5 months (interquartile range 2.0-4.4). The most common new symptoms ≥4 weeks after a diagnosis of covid-19 were joint pain (2.5% of patients), abdominal pain (0.9%), and headache (0.8%). In adjusted analyses, compared with 12 months previously, GP consultation rates for patients with covid-19 managed in the community were higher after covid-19 for loss of smell or taste, or both (adjusted hazard ratio 5.28, 95% confidence interval 3.89 to 7.17, P<0.001), muscle pain (1.89, 1.63 to 2.20, P<0.001), fatigue (1.64, 1.53 to 1.76, P<0.001), insomnia (1.50, 1.33 to 1.69, P<0.001), and palpitations (1.42, 1.27 to 1.59, P<0.001). GP consultation rates for breathlessness, tinnitus, paraesthesia, and chest pain were also higher but to a lesser extent. Joint pain, sore throat, cough, fever, and skin rashes were significantly lower after compared with before covid-19 (fig 2, and tables S2 and S7).

Among this cohort, anxiety (1.2% of patients), depression (0.9%), and asthma (0.7%) were the most common new onset diseases ≥4 weeks after covid-19. Compared with 12 months before infection, GP consultation rates for venous thromboembolism (3.35, 2.87 to 3.91, P<0.001), lung fibrosis (2.41, 1.37 to 4.25, P=0.002), renal failure (1.33, 1.17 to 1.52, P<0.001), and diabetes (1.13, 1.06 to 1.21, P<0.001) were higher after covid-19. For patients with covid-19 in the community, GP consultation rates for asthma, arthritis, and hypertension were significantly lower after covid-19 compared with the period 12 months previously (fig 2, and tables S3 and S7). The most common prescriptions ≥4 weeks after covid-19 were for non-steroidal anti-inflammatory drugs (1.2% of patients), opioids (1.0%), and bronchodilators (0.8%). Patients in the community were more likely to receive strong opiates (adjusted hazard ratio 1.18, 95% confidence interval 1.07 to 1.31, P=0.001) and neuropathic pain drugs (1.15, 1.08 to 1.23, P<0.001) after covid-19 than 12 months previously. Prescription rates for bronchodilators and inhaled corticosteroids were significantly lower after covid-19 compared with rates 12 months before infection (fig 2, and tables S4 and S7). After covid-19, patients in the community had higher rates of healthcare use overall (1.15, 1.14 to 1.15, P<0.001), admissions to hospital (1.16, 1.06 to 1.26, P<0.001), and visits to primary care (1.18, 1.17 to 1.19, P<0.001) compared with 12 months before the diagnosis of covid-19. The rate of outpatient referrals was significantly lower after covid-19 compared with 12 months before infection (fig 3, and tables S5 and S7).

### Contextualising outcomes pre-covid-19 versus post-covid-19 using negative control and influenza cohorts

A total of 38 511 patients were included in the negative control group and 21 803 in the influenza cohort (table S8 and fig S1). Healthcare use before and after the index dates in patients with covid-19 managed in the community and the negative control populations were comparable but we found some differences. Relative to the negative control and influenza cohorts, GP consultation rates were significantly higher after covid-19 for loss of smell or taste, or both, fatigue, palpitations, breathlessness, tinnitus, paraesthesia, chest pain, muscle pain, lung fibrosis, venous thromboembolism, and renal failure in patients in the community. In patients in the community, we found fewer diagnoses of ischaemic heart disease, hypertension, asthma, and arthritis after than before covid-19 infection, but only fewer diagnoses for asthma in the negative control cohort. We found an increase in diagnoses of anaemia after acute infection only in the influenza cohort (tables S7, S9, and S10).

### Incidence of outcomes for each month after covid-19 in patients managed in the community

**Fig 4:**
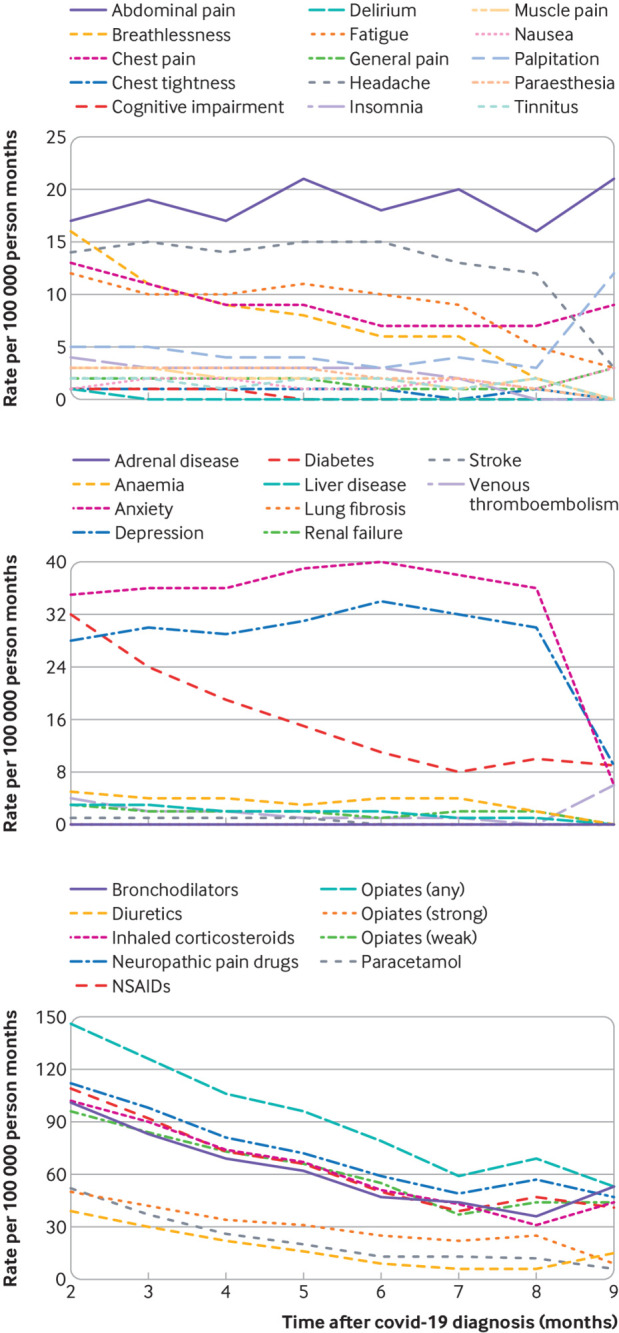
Crude general practitioner consultation rates for symptoms, diseases, and drug prescriptions for each month after a covid-19 diagnosis in patients managed in the community. Only diseases and symptoms that had a significant increase in rate of occurrence after covid-19 were included. All drug prescription outcomes were included, regardless of whether they were significantly associated with an increase after covid-19. NSAIDs=non-steroidal anti-inflammatory drugs

GP consultation rates that persisted after covid-19 in patients in the community included abdominal pain and, to a lesser extent, diarrhoea, muscle pain, general pain, tinnitus, nausea, and chest tightness (fig 4). In contrast, breathlessness, headache, chest pain, and fatigue declined over time. Anxiety and depression and, to some extent, lung fibrosis, were generally constant from month to month, whereas the rate of diabetes, anaemia, venous thromboembolism, and stroke declined over time. The rate of all drug prescriptions declined over follow-up.

### Covid-19 outcomes before versus after vaccination in patients managed in the community

**Fig 5:**
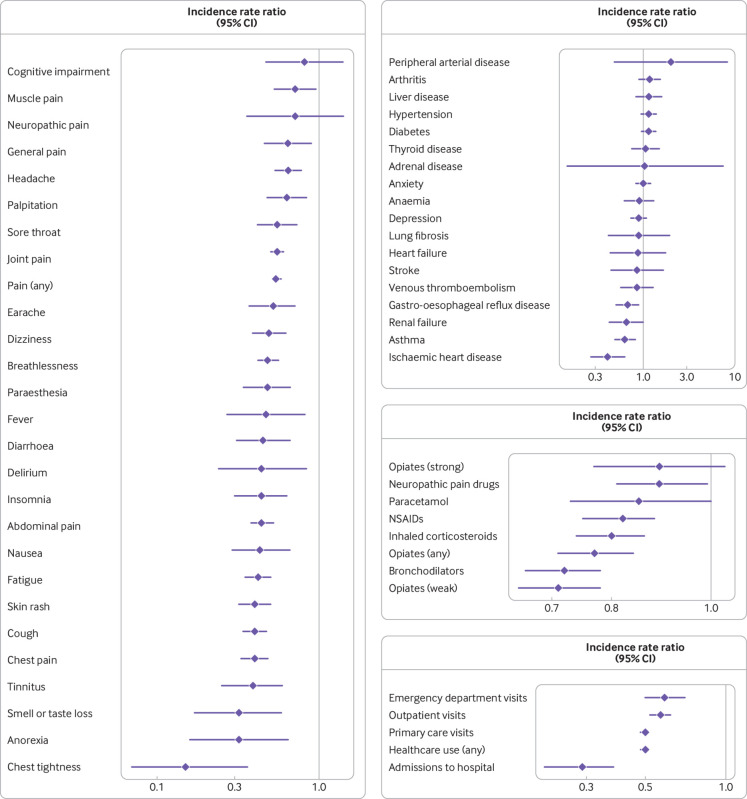
General practitioner consultation rates for symptoms, diseases, drug prescriptions, and healthcare use after a covid-19 diagnosis in patients managed in the community who received a vaccine dose for covid-19 after diagnosis. Forest plots show incidence rate ratios (95% confidence interval) adjusted for age, sex, Charlson comorbidity index, body mass index, smoking, and time since covid-19 diagnosis for all outcomes, excluding venous thromboembolism, paraesthesia, and palpitations where adjustment for body mass index was not possible because of low number of events. Irritable bowel disease is not shown because of 0 events recorded during follow-up. NSAIDs=non-steroidal anti-inflammatory drugs

Of those individuals who had a covid-19 diagnosis and managed in the community (n=437 943), 267 993 (61.2%) were censored at the date of their first covid-19 vaccination. Of these, 19 151 (7.1%) patients reported at least one symptom after covid-19 infection before vaccination. GP consultation rates after versus before vaccination were reduced for chest tightness (adjusted incidence rate ratio 0.15, 95% confidence interval 0.07 to 0.36, P<0.001); anorexia (0.32, 0.16 to 0.64, P=0.001); loss of smell or taste, or both (0.32, 0.17 to 0.58, P=0.002); tinnitus (0.39, 0.25 to 0.59, P<0.001); and chest pain (0.40, 0.33 to 0.48, P<0.001). Patients also had reduced GP consultation rates for all other symptoms after vaccination, except neuropathic pain and cognitive impairment (fig 5, and tables S11 and S15). GP consultation rates after vaccination were reduced for ischaemic heart disease (adjusted incidence rate ratio 0.41, 95% confidence interval 0.27 to 0.63, P<0.001), asthma (0.63, 0.49 to 0.82, P<0.001), and gastro-oesophageal reflux disease (0.68, 0.51 to 0.89, P=0.006) (fig 5, and tables S12 and S15). In contrast, rates for prescriptions for all investigated drugs, except strong opiates and paracetamol, were significantly lower after than before vaccination (fig 5, and tables S13 and S15). Patients with covid-19 managed in the community who received at least one vaccine dose also seemed to have lower rates for use of all healthcare resources (adjusted incidence rate ratio 0.50, 95% confidence interval 0.48 to 0.51, P<0.001), admissions to hospital (0.29, 0.21 to 0.38, P<0.001), and visits to primary care (0.50, 0.48 to 0.51, P<0.001) and the emergency department (0.59, 0.50 to 0.70, P<0.001) after vaccination (fig 5, and tables S14 and S15).

### Sensitivity analysis

Results were similar when we classified patients as admitted to hospital after three or four weeks after testing positive for covid-19 or after two weeks. The number of patients classed as admitted to hospital within three weeks of a positive test was 20 870 (4.58%) and 22 645 (4.97%) within four weeks of a positive test. The median interval between a diagnosis of covid-19 and smoking status was 1.4 years (interquartile range 0.5-3.1). The number of patients with a recorded body mass index within one and two years of the index date was 22.4% and 42.2%, respectively, compared with 66.3% of patients with a body mass index recorded within five years of the index date (page 3, supplementary material). Analyses of GP consultation rates for symptom and prescription outcomes from two weeks after a diagnosis of covid-19 were similar to a four week window (tables S16 and S17). Re-running Cox regression analyses without exclusion of patients with a history of an outcome of interest before the index date gave similar results to the main analysis (table S18). Lastly, we compared rates one month before with rates after vaccination in the vaccination cohort, and found similar findings to the main vaccine analyses before and after vaccination (table S19).

## Discussion

### Principal findings

We found that patients with covid-19 admitted to hospital and those managed in the community had higher GP consultation rates for most symptoms and diseases, received more prescriptions, and were more likely to use healthcare resources after covid-19 than in the 12 months before infection, although the rates in the two groups differed. For example, although the rates for primary care consultations for symptoms such as fatigue, breathlessness, and palpitations were similar between the two groups, patients in the community were more likely to consult their GP because of loss of taste and smell and muscle pain; patients admitted to hospital were more likely to report ongoing problems related to nausea and delirium.

Although healthcare use increased in both groups after covid-19 relative to levels before the pandemic, the increase was higher in patients admitted to hospital for all types of healthcare use. Nevertheless, healthcare use in the group managed in the community increased by 18% after covid-19 compared with levels before the pandemic, highlighting the need for adequate ongoing provision of care for this population. We also found that some outcomes improved after vaccination in the cohort managed in the community, giving hope that with time and increased vaccination rates most sequelae will resolve. We also saw a decrease over time in drug prescriptions, in particular for bronchodilators. Whether fewer prescriptions was a consequence of lack of improvement in symptoms with bronchodilators or increased recognition of dysfunctional breathing after covid-19 needs further investigation.

### Comparison with related studies

According to the Office for National Statistics,^24^ just over one million people in the UK (13% of those who had covid-19) had persistent symptoms at least four weeks after covid-19 infection, and in 65.9% of individuals, symptoms were adversely affecting their day-to-day activities. The REACT-2 (real time assessment of community transmission 2) study^25^ showed even higher estimates, noting that a third of people in England who had covid-19 developed long term symptoms (>12 weeks), with an estimated two million people affected. We found that 8.5% of the cohort with covid-19 managed in the community had ongoing symptom related sequelae, with some reduction in symptom burden after vaccination. Moreover, whereas REACT-2 found that the most common persistent symptoms were tiredness, shortness of breath, muscle aches, and difficulty sleeping, we found that the commonest symptoms were joint pain, breathlessness, and cough in those admitted to hospital, and abdominal pain, joint pain, and headache in those managed in the community. Differences between the Office for National Statistics and REACT-2 studies and ours are likely because the former relied on survey data whereas we used routine data from electronic healthcare records. Patients with ongoing symptoms after acute covid-19 infection might not visit their GP for some symptoms or might find it difficult to access healthcare services,^16^ ^26^ and therefore the true burden of these particular symptoms might not be fully captured in primary care records. Equally, those who respond to surveys might be more likely to be symptomatic and thus overestimate outcomes. Compared with our study, which analysed patients with a confirmed diagnosis of covid-19, the survey by the Office for National Statistics also included respondents with suspected infection. Other studies have reported substantial heterogeneity in the range of symptoms reported after covid-19.^13^ ^17^ ^27^ ^28^ This multisystem impairment was also a feature of our study; patients in the community had higher rates of developing venous thromboembolism, lung fibrosis, and renal failure after covid-19 relative to the 12 months before infection.

A Danish population based study of patients managed in the community found that individuals were more likely to visit their GP or attend a hospital outpatient clinic but were not more likely to be admitted to hospital.^18^ This result contrasts with our finding of increased use of healthcare resources in patients in the community after covid-19 in the UK, with respect to visits to primary care and admissions to hospital. This increase since before the pandemic suggests that healthcare access during the second wave was less difficult than during the first wave, but evidence indicates that accessing healthcare services can nevertheless still be difficult.^14^

Although we used individuals in each cohort as their own historical controls by comparing outcome rates after covid-19 and 12 months before infection, we looked at the issue of temporality (that is, whether outcomes after covid-19 were caused by the acute infection or confounded by other temporal aspects of the pandemic) by studying a negative control cohort for patients with covid-19 managed in the community, similar to previous studies.^17^ ^27^ Also, sequelae after viral infections are common and therefore we included an historical influenza cohort (before the covid-19 pandemic) to determine whether outcomes after covid-19 were related to acute viral respiratory infection rather than covid-19 itself.^12^ ^13^ We found that healthcare use in the covid-19 cohort in the community was similar to that in the negative control group, indicating that access to healthcare was similar in both groups. In contrast, compared with the negative control and influenza cohorts, several outcomes were more frequent after covid-19 infection, including symptoms such as loss of smell or taste, or both, fatigue, breathlessness, tinnitus, palpitations, paraesthesia, muscle pain, and chest pain, and diseases such as lung fibrosis, venous thromboembolism, and renal failure. This finding suggests that some sequelae after acute covid-19 infection are specific to covid-19 rather a post-viral syndrome. Reduced GP consultation rates after versus before covid-19 infection for joint pain, cough, sore throat, fever, and skin rash might be because these symptoms are more commonly associated with acute infection.

We found that GP consultation rates for diagnoses of ischaemic heart disease, hypertension, asthma, and arthritis were lower after than before covid-19 infection, relative to the negative control and influenza cohorts. Studies in patients admitted to hospital have shown that those with covid-19 are more likely to have had cardiovascular diseases (such as hypertension) before the acute infection than those with influenza, which are associated with worse outcomes related to covid-19, and that the mortality rate is higher in covid-19 than in influenza.^29^ Both of these factors could have contributed to the lower rates of new cardiovascular diseases, such as ischaemic heart disease or hypertension, after covid-19 relative to those seen in the other two groups. Other contributing factors could be that symptoms such as arthralgia or chest pain were attributed to long covid rather than other diseases. Furthermore, the use of routine diagnostics during the pandemic was limited. For instance, diagnoses of airways diseases, such as asthma, might have been hampered by lack of diagnostics over this period because of suspension of spirometry and pulmonary function testing in primary and secondary care owing to concerns about generation of aerosols. Fewer new diagnoses of asthma might also have been because breathlessness or wheeze was attributed to other causes, such as sequelae after acute covid-19 infection, or to social distancing, and lockdown measures, resulting in less transmission of other respiratory viruses and therefore asthma flares; changes in healthcare seeking behaviour in people with chronic breathing problems might also have been a factor.^30^ Because of social distancing measures, outpatient and primary care clinical reviews changed from face-to-face to remote access, and so chest examinations were often not possible. Similarly, individuals with symptoms such as cough were advised not to attend primary care clinics during the pandemic, which could partially explain the decreased rates of recorded cough and sore throat after covid-19 compared with rates 12 months before infection.

In contrast, outcomes for symptoms, diseases, and prescriptions before and after influenza infection were similar, which could be because influenza is a common yearly endemic infection with a well known disease profile, epidemiology, and preventive vaccination programme. Patients with influenza might be less likely to seek healthcare services, and if they do, clinicians tend to manage symptoms more conservatively. Alternatively, our findings could be because influenza is less commonly associated with multisystem complications, such as venous thromboembolism, than covid-19, as has been shown in previous studies.^30^

Our findings suggest that vaccination could help reduce the burden of sequelae after covid-19 infection, at least in patients in the community with ongoing symptoms after the acute infection. Although limited information exists for vaccination and long covid, data from a survey of 900 people reported in the Long Covid SOS report^19^ support our findings. Similar results were found in a UK study comparing 44 patients vaccinated after admission to hospital^31^ and in a study analysing data from the Covid Symptom Study app.^32^ In our study, we found that as well as improving symptoms, vaccination can help reduce the incidence of ischaemic heart disease, asthma, and gastro-oesophageal reflux disease, and reduce the need for analgesics and inhalers and overall healthcare use, at least in patients in the community with persistent symptoms after acute covid-19. Our analyses were however limited to the first covid-19 vaccine dose and therefore further studies are needed to characterise the association between vaccination and sequelae after covid-19 in patients given a full vaccination course.

Our analysis of temporal trends in sequelae after acute covid-19 in patients in the community suggests that the nature of long covid might be dynamic, with temporal changes in several outcomes. In addition to temporal changes in symptoms, diseases, and drug use after covid-19, some studies suggest a similar phenomenon for healthcare use.^33^ ^34^ Changes in several outcomes could be because of more active monitoring of patients close to the time of the diagnosis of covid-19.

### Strengths and limitations

Our study included patients with covid-19 from the second wave of the pandemic in the UK, when testing capacity was much higher, and therefore potential selection biases were limited. Although we cannot determine whether symptoms recorded in primary care were directly caused by covid-19, we compared GP consultation rates for the same two covid-19 cohorts (those admitted to hospital and those in the community) 12 months before covid-19, separately, to contextualise our findings. We also compared our outcomes in negative controls and in an historical cohort of patients with another viral respiratory infection. A window for exclusion of outcome events after a diagnosis of covid-19 also ensured that the outcomes were not related to the infection itself. We minimised the risk of misclassification of outcomes by the use of previously validated code lists wherever possible and creating code lists tailored to the objectives of this study. We recognise, however, that recording of symptoms is dependent on data entry and coding by GPs, which might have introduced bias.

Our study had some limitations. We could not determine from the data available whether patients were symptomatic during infection. We also could not capture the effect of socioeconomic status because these data are not available in primary care records. We could not consider the severity of outcomes of covid-19 infection.

Given the relatively short follow-up period of a maximum of nine months, outcome events which occur later in the course of long covid would not have been captured. We plan to repeat the analyses with longer follow-up in future studies, however. We probably did not capture outcomes after acute covid-19 in patients who did not seek medical care and managed symptoms independently with over-the-counter drugs. Furthermore, patients were excluded from Cox regression analyses if they had the outcome of interest one year before the index dates and this exclusion could have biased the results. Sensitivity analyses that did not exclude patients, however, showed similar results to our main findings. We found a high level of missingness for body mass index, a covariate, but the five year window before the index date that we used to capture as many patients as possible might have introduced some information bias. The negative control group could have included false negatives and might explain why outcomes in this population were similar to the covid-19 cohort managed in the community. Lastly, the CPRD Aurum is limited to individuals in England and findings might not apply to patients from other countries.

### Implications of our findings

With more than eight million individuals recovered from acute covid-19 in the UK at the time of writing,^11^ our findings of multisystem symptom and disease burden have substantial implications for future planning of healthcare services and highlight the importance of providing integrated multidisciplinary care in the management of patients after covid-19 infection. Although healthcare use increased relatively more in those admitted to hospital, we also found an increase in those managed in the community, suggesting that current provision of healthcare services might need to be changed to meet this increasing demand. Furthermore, findings from this study are timely given the substantial interest in long covid globally and calls for further research in assessing interventions in individuals in the community who continue to have sequelae of acute covid-19. 

Increased awareness among clinicians of the dynamic nature of the burden of some symptoms and diseases after acute covid-19, at least in those in the community, is needed to help provide adequate support and management of patients after the acute infection. Finally, we found a reduced incidence of symptoms and diseases after vaccination for covid-19 although we only analysed outcomes in patients with covid-19 in the community. Hence our study lends support to the ongoing public health campaign to promote covid-19 vaccination, not only to reduce the risk of acute infection but also to minimise the risk of longer term complications after covid-19.

### Conclusions

GP consultation rates for sequelae after acute covid-19 infection were different in patients admitted to hospital for covid-19 and those managed in the community. In individuals in the community, some sequelae decreased over time and with vaccination, but others such as anxiety and depression persisted. Recognition of the different profiles of sequelae in each group and their dynamic nature is important in guiding appropriate care for patients after covid-19 infection.

**What is already known on this topic**

Persistent symptoms and new organ dysfunction after acute covid-19 infection have been recognised in several observational studies but these findings have been primarily seen in patients admitted to hospital with more severe disease
Few studies have compared long term outcomes in individuals with covid-19 managed in the community, with most studies limited by small cohort sizes and selection biases
No large population based cohort studies have assessed outcomes over time or after vaccination for covid-19

**What this study adds**

General practitioner consultation rates for sequelae after acute covid-19 infection differed between patients admitted to hospital with covid-19 and those managed in the community
Patients with covid-19 managed in the community had higher consultation rates for multiple sequelae, which were more prevalent than consultation rates after viral respiratory infections, such as influenza; the most common sequelae were loss of smell or taste, or both, venous thromboembolism, lung fibrosis, and muscle pain, and also increased use of healthcare services compared with 12 months before infection
A small proportion (8.5%) of patients in the community had ongoing symptom related sequelae after acute covid-19, with some reduction in symptom burden after vaccination

## Data Availability

Linked pseudonymised mortality data from the Office for National Statistics (ONS), socioeconomic data from the index of multiple deprivation (IMD), and secondary care data from Hospital Episode Statistics (HES) were provided for this study by the Clinical Practice Research Datalink (CPRD) for patients in England. Data are linked by NHS Digital, the statutory trusted third party for linking data, with identifiable data held only by NHS Digital. Select general practices consent to this process at a practice level, with individual patients having the right to opt out. Use of HES and ONS data are copyright (2018), re-used with the permission of the Health and Social Care Information Centre, all rights reserved. Data are available on request from the CPRD. Their provision requires the purchase of a license, and this license does not permit the authors to make them publicly available to all. This work used data from the version collected in January 2021 and have clearly specified the data selected in the methods section. To allow identical data to be obtained by others, via the purchase of a license, the code lists have been provided on GitHub. Licenses are available from the CPRD ( This study used existing data from the UK Clinical Practice Research Datalink (CPRD) electronic health record database; this data resource is accessible only to researchers with protocols approved by the CPRD’s independent scientific advisory committee and, therefore, no additional unpublished data are available. All data management and analysis computer code are available on request. The study protocol and analysis plan are available in the supplementary material.https://www.cprd.com), The Clinical Practice Research Datalink Group, the Medicines and Healthcare products Regulatory Agency, 10 South Colonnade, Canary Wharf, London E14 4PU.

## Web extra

Extra material supplied by authors

Web appendix: Supplementary material

## Notes

Contributors: HRW, CG, and AK are joint first authors. JKQ conceptualised the study and all authors contributed to study design. HRW, CG, AK, ADM, CI, and RG created code lists for outcomes of interest. HRW, CG, CK, and ADM prepared the data and performed statistical analyses, with all authors contributing to interpretation of results. Figures were created by CG and HRW. AK, HRW, ADM, CG, and JKQ drafted the original manuscript, with critical revision of the manuscript by all authors. All authors approved the final manuscript. The corresponding author attests that all listed authors meet authorship criteria and that no others meeting the criteria have been omitted. The corresponding author is also the guarantor for this manuscript and accepts full responsibility for the work, had access to all the data, and was responsible for the decision to publish.

The corresponding author confirms that the manuscript is an honest, accurate, and transparent account of the study being reported, that no important aspects of the study have been omitted, and that any discrepancies from the study as originally planned have been explained.

Dissemination to participants and related patient and public communities: We will share results and methodology with other researchers with these and similar data to answer questions on long term consequences of covid-19. We will distribute results via BREATHE and the wider Health Data Research (HDR) UK consortium, including charities such as AUK-BLF (Asthma UK and British Lung Foundation Partnership). We will also prepare a press release via Imperial College, add a plain language summary to the HDR UK BREATHE data hub website, and tweet about the findings to maximise knowledge distribution to clinicians and the public.

Provenance and peer review: Not commissioned; externally peer reviewed.

## References

[1] Puntmann VO Carerj ML Wieters I . Outcomes of cardiovascular magnetic resonance imaging in patients recently recovered from coronavirus disease 2019 (COVID-19). JAMA Cardiol 2020;5:1265-73. 10.1001/jamacardio.2020.3557 32730619 PMC7385689

[2] Fraser E . Long term respiratory complications of covid-19. BMJ 2020;370:m3001. 10.1136/bmj.m3001 32747332

[3] Rubino F Amiel SA Zimmet P . New-onset diabetes in COVID-19. N Engl J Med 2020;383:789-90. 10.1056/NEJMc2018688 32530585 PMC7304415

[4] Di Minno A Ambrosino P Calcaterra I Di Minno MND . COVID-19 and venous thromboembolism: a meta-analysis of literature studies. Semin Thromb Hemost 2020;46:763-71. 10.1055/s-0040-1715456 32882719 PMC7645842

[5] Taquet M Luciano S Geddes JR Harrison PJ . Bidirectional associations between COVID-19 and psychiatric disorder: retrospective cohort studies of 62 354 COVID-19 cases in the USA. Lancet Psychiatry 2021;8:130-40. 10.1016/S2215-0366(20)30462-4 33181098 PMC7820108

[6] Paterson RW Brown RL Benjamin L . The emerging spectrum of COVID-19 neurology: clinical, radiological and laboratory findings. Brain 2020;143:3104-20. 10.1093/brain/awaa240 32637987 PMC7454352

[7] National Institute for Health and Care Excellence. COVID-19 rapid guideline: managing the long-term effects of COVID-19. 2021. https://www.nice.org.uk/guidance/ng188/resources/covid19-rapid-guideline-managing-the-longterm-effects-of-covid19-pdf-51035515742 33555768

[8] World Health Organization. A clinical case definition of post COVID-19 condition by a Delphi consensus, 6 October 2021. 2021. https://www.who.int/publications/i/item/WHO-2019-nCoV-Post_COVID-19_condition-Clinical_case_definition-2021.1.

[9] Lopez-Leon S Wegman-Ostrosky T Perelman C . More than 50 long-term effects of COVID-19: a systematic review and meta-analysis. medRxiv 2021. 10.1101/2021.01.27.21250617 PMC8352980 34373540

[10] Knock E, Whittles L, Lees J, et al. Report 41: The 2020 SARS-CoV-2 epidemic in England: key epidemiological drivers and impact of interventions. 2020, Imperial College London. 10.25561/85146 https://www.imperial.ac.uk/mrc-global-infectious-disease-analysis/covid-19/report-41-rtm/ PMC8432953 34158411

[11] UK government. Coronavirus (Covid-19) dashboard https://coronavirus.data.gov.uk/details/healthcare.

[12] Al-Aly Z Xie Y Bowe B . High-dimensional characterization of post-acute sequelae of COVID-19. Nature 2021;594:259-64. 10.1038/s41586-021-03553-9 33887749

[13] Daugherty SE Guo Y Heath K . Risk of clinical sequelae after the acute phase of SARS-CoV-2 infection: retrospective cohort study. BMJ 2021;373:n1098. 10.1136/bmj.n1098 34011492 PMC8132065

[14] Alwan NA Attree E Blair JM . From doctors as patients: a manifesto for tackling persisting symptoms of covid-19. BMJ 2020;370:m3565. 10.1136/bmj.m3565 32933949

[15] Goërtz YMJ Van Herck M Delbressine JM . Persistent symptoms 3 months after a SARS-CoV-2 infection: the post-COVID-19 syndrome?. ERJ Open Res 2020;6:00542-2020. 10.1183/23120541.00542-2020 33257910 PMC7491255

[16] Ladds E Rushforth A Wieringa S . Persistent symptoms after Covid-19: qualitative study of 114 “long Covid” patients and draft quality principles for services. BMC Health Serv Res 2020;20:1144. 10.1186/s12913-020-06001-y 33342437 PMC7750006

[17] Augustin M Schommers P Stecher M . Post-COVID syndrome in non-hospitalised patients with COVID-19: a longitudinal prospective cohort study. Lancet Reg Health Eur 2021;6:100122. 10.1016/j.lanepe.2021.100122 34027514 PMC8129613

[18] Lund LC Hallas J Nielsen H . Post-acute effects of SARS-CoV-2 infection in individuals not requiring hospital admission: a Danish population-based cohort study. Lancet Infect Dis 2021;21:1373-82. 10.1016/S1473-3099(21)00211-5 33984263 PMC8110209

[19] Strain WD Sherwood O Banerjee A . The Impact of COVID Vaccination on Symptoms of Long COVID. An International Survey of People with Lived Experience of Long COVID. SSRN 2021. 10.3390/vaccines10050652 PMC9146071 35632408

[20] Medicines and Healthcare products Regulatory Agency. Release note CPRD Aurum. 2021. https://www.cprd.com/sites/default/files/2021-06%20CPRD%20Aurum%20Release%20Notes.pdf.

[21] Wolf A Dedman D Campbell J . Data resource profile: clinical practice research datalink (CPRD) aurum. Int J Epidemiol 2019;48:1740-1740g. 10.1093/ije/dyz034 30859197 PMC6929522

[22] Metcalfe D Masters J Delmestri A . Coding algorithms for defining Charlson and Elixhauser co-morbidities in Read-coded databases. BMC Med Res Methodol 2019;19:115. 10.1186/s12874-019-0753-5 31170931 PMC6554904

[23] Rogers W . Regression standard errors in clustered samples. Stata Tech Bull 1994;3.

[24] Ayoubkhani D, Pawelek P, Bosworth M. Prevalence of ongoing symptoms following coronavirus (COVID-19) infection in the UK: 5 August 2021. 2021. https://www.ons.gov.uk/peoplepopulationandcommunity/healthandsocialcare/conditionsanddiseases/bulletins/prevalenceofongoingsymptomsfollowingcoronaviruscovid19infectionintheuk/5august2021

[25] O’Dowd A . Covid-19: Third of people infected have long term symptoms. BMJ 2021;373:n1626. 10.1136/bmj.n1626 34168002

[26] Moynihan R Sanders S Michaleff ZA . Impact of COVID-19 pandemic on utilisation of healthcare services: a systematic review. BMJ Open 2021;11:e045343. 10.1136/bmjopen-2020-045343 33727273 PMC7969768

[27] Ayoubkhani D Khunti K Nafilyan V . Post-covid syndrome in individuals admitted to hospital with covid-19: retrospective cohort study. BMJ 2021;372:n693. 10.1136/bmj.n693 33789877 PMC8010267

[28] Salamanna F Veronesi F Martini L Landini MP Fini M . Post-COVID-19 syndrome: the persistent symptoms at the post-viral stage of the disease. a systematic review of the current data. Front Med (Lausanne) 2021;8:653516. 10.3389/fmed.2021.653516 34017846 PMC8129035

[29] Piroth L Cottenet J Mariet A-S . Comparison of the characteristics, morbidity, and mortality of COVID-19 and seasonal influenza: a nationwide, population-based retrospective cohort study. Lancet Respir Med 2021;9:251-9. 10.1016/S2213-2600(20)30527-0 33341155 PMC7832247

[30] Doe G Chantrell S Williams M . Breathless and awaiting diagnosis in UK lockdown for COVID-19…We’re stuck. NPJ Prim Care Respir Med 2021;31:21. 10.1038/s41533-021-00232-0 33953200 PMC8100135

[31] Arnold DT Milne A Stadon L . Are vaccines safe in patients with long COVID? A prospective observational study. medRxiv 2021. 10.1101/2021.03.11.21253225 .

[32] Antonelli M Penfold RS Merino J . Risk factors and disease profile of post-vaccination SARS-CoV-2 infection in UK users of the COVID Symptom Study app: a prospective, community-based, nested, case-control study. Lancet Infect Dis 2021;S1473-3099(21)00460-6. 10.1016/S1473-3099(21)00460-6. 34480857 PMC8409907

[33] Hernandez-Romieu AC Leung S Mbanya A . Health care utilization and clinical characteristics of nonhospitalized adults in an integrated health care system 28-180 days after COVID-19 diagnosis - Georgia, May 2020-March 2021. MMWR Morb Mortal Wkly Rep 2021;70:644-50. 10.15585/mmwr.mm7017e3 33914727 PMC8084119

[34] Skyrud KD Telle KE Magnusson K . Impacts of COVID-19 on long-term health and health care use. medRxiv 2021 10.1101/2021.02.16.21251807 .

[35] UK government. Coronavirus. (Covid-19) dashboard https://coronavirus.data.gov.uk/details/cases.

