# Supplementary material for: GP consultation rates for sequelae after acute covid-19 in patients managed in the community or hospital in the UK: population based study
Source: BMJ. 2021 Dec 29;375:e065834. doi: 10.1136/bmj-2021-065834 (PMC8715128; doi:10.1136/bmj-2021-065834)
Supplement: Supplementary file 1 — Web appendix: Supplementary material [file whih065834.ww.pdf]

<u>Post-acute COVID-19 sequelae in cases admitted to hospital and not admitted to hospital in the UK: a population based study</u>

Supplementary material

Contents

Defining BMI using 1, 2, and 5 years from start of follow-up 3

Table S1: Frequency of patients by geographic location in England 4

Table S2: Symptom event rates (per 100,000 person-weeks) 5

Table S3: Disease event rates (per 100,000 person-weeks). 9

Table S4: Prescription event rates (per 100,000 person-weeks). 13

Table S5: Health care utilization (HCU) event rates (per 100,000 person-weeks). 15

Table S6: Hazard ratios (95% CI) for differences in outcome event rates between patients hospitalised with COVID-19 and the same patients 12 months prior to COVID-19 diagnosis. 16

Bonferroni significance threshold for symptoms=0.002, for diseases=0.003, for medications=0.005, and for HCU=0.01. *adjusted for age, sex, smoking status, BMI, and CCI. 18

Table S7: Hazard ratios (95% CI) for differences in outcome event rates between patients with Community COVID-19 and the same patients 12 months prior to COVID-19 diagnosis. 19

Table S8: Baseline characteristics for negative control and influenza cohorts 22

Table S9: Hazard ratios (95% CI) for differences in outcome event rates between patients in the negative control group and the same patients 12 months prior. 23

Table S10: Hazard ratios (95% CI) for differences in outcome event rates between patients with influenza and the same patients 12 months prior to having influenza. 26

Table S11: Event rates (per 100,000 person-weeks) for Community COVID-19 patients in the period from COVID-19 diagnosis to vaccination and from vaccination to end of follow-up. 29

Table S12: Event rates (per 100,000 person-weeks) for Community COVID-19 patients in the period from COVID-19 diagnosis to vaccination and from vaccination to end of follow-up. 30

Table S13: Event rates (per 100,000 person-weeks) for Community COVID-19 patients in the period from COVID-19 diagnosis to vaccination and from vaccination to end of follow-up. 32

Table S14: Event rates (per 100,000 person-weeks) for Community COVID-19 patients in the period from COVID-19 diagnosis to vaccination and from vaccination to end of follow-up. 33

Table S15: IRR (95% CI) for differences in outcome event rates between symptomatic, vaccinated patients with Community COVID-19 and the same patients after vaccination. 34

Table S16: Symptom event rates (per 100,000 person-weeks) for 2-week washout sensitivity analysis.. 37

Table S17: Prescription event rates (per 100,000 person-weeks) for 2-week washout sensitivity analysis. 42

Table S18: Hazard ratios (95% CI) for differences in outcome event rates between patients with COVID-19 and the same patients 12 months prior to having COVID-19 and not excluding patients with previous events. 44

Table S19: IRR (95% CI) for differences in outcome event rates between symptomatic, vaccinated patients with Community COVID-19 one month prior to vaccination and the same patients after vaccination. 46

Figure S1: Inclusion and exclusion criteria for each cohort: Community COVID-19 cohort, hospitalised COVID-19 cohort, Community vaccination cohort, Community negative control cohort, and Community influenza cohort. 49

List of abbreviations

IHD - Ischemic Heart Disease
PAD - Peripheral Arterial Disease
VTE - Venous Thromboembolism
GORD - Gastro-Oesophageal Reflux Disease
ICS - Inhaled Corticosteroids
NSAIDS - Non-Steroidal Anti-Inflammatory Drugs

Defining BMI using 1, 2, and 5 years from start of follow-up

BMI category	Measurement within 1 year of start of follow-up	Measurement within 2 years of start of follow-up	Measurement within 5 years of start of follow-up
Underweight	2,173 (0.5)	4,025 (0.9)	6,733 (1.5)
Normal	27,631 (6.1)	56,056 (12.3)	95,531 (210.)
Overweight	31,586 (6.9)	61,369 (13.5)	99,958 (21.9)
Obese	40,723 (8.9)	71,026 (15.6)	101,022 (22.2)
Missing	353,889 (77.6)	263,513 (57.8)	152,758 (33.5)

Table S1: Frequency of patients by geographic location in England

Geographic location in England	Frequency of patients n(%) N=456,002
**North east**	15,431 (3.4)
**North west**	100,844 (22.1)
**Yorkshire and the Humber**	13,172 (2.9)
**East Midlands**	11,205 (2.5)
**West Midlands**	75,825 (16.6)
**East**	15,950 (3.5)
**South west**	34,788 (7.6)
**South central**	42,637 (9.4)
**London**	106,121 (23.3)
**South east coast**	39,334 (8.6)
**Unknown**	695 (0.2)

Table S2: Symptom event rates (per 100,000 person-weeks)

Outcome	COVID exposure group	Time point	Number of events	% of patients with outcome	Time (person-weeks (PW))	Rate (per 100,000 PW)	Lower CI	Upper CI
General pain	Community	12-months before	428	0.10	65.5	6.5	5.9	7.2
		AfterCOVID-19	518	0.12	65.5	7.9	7.3	8.6
	Hospital	12-months before	27	0.15	2.0	13.8	9.6	20.6
		AfterCOVID-19	36	0.20	2.0	18.4	13.4	26.0
Chest pain	Community	12-months before	2106	0.48	65.3	32.3	30.9	33.7
		AfterCOVID-19	2699	0.62	65.2	41.4	39.9	43.0
	Hospital	12-months before	141	0.78	1.9	72.7	61.9	86.1
		AfterCOVID-19	208	1.15	1.9	108.6	95.0	124.7
Abdominal pain	Community	12-months before	3740	0.85	65.0	57.5	55.7	59.4
		AfterCOVID-19	4124	0.94	65.0	63.5	61.6	65.4
	Hospital	12-months before	152	0.84	1.9	78.5	67.2	92.4
		AfterCOVID-19	183	1.01	1.9	95.8	83.1	111.0
Headache	Community	12-months before	3042	0.69	65.1	46.7	45.1	48.4
		AfterCOVID-19	3317	0.76	64.9	51.1	49.4	52.9
	Hospital	12-months before	106	0.59	1.9	54.5	45.2	66.3
		AfterCOVID-19	92	0.51	1.9	48.0	39.3	59.3
Joint pain	Community	12-months before	11611	2.65	63.7	182.3	179.0	185.6
		AfterCOVID-19	11065	2.53	63.9	173.3	170.1	176.5
	Hospital	12-months before	565	3.13	1.9	301.0	277.5	327.0
		AfterCOVID-19	495	2.74	1.9	266.3	244.2	290.9
Muscle pain	Community	12-months before	335	0.08	65.5	5.1	4.6	5.7
		AfterCOVID-19	613	0.14	65.5	9.4	8.7	10.1
	Hospital	12-months before	20	0.11	2.0	10.2	6.7	16.4
		AfterCOVID-19	43	0.24	2.0	22.0	16.5	30.2
Neuropathic pain	Community	12-months before	97	0.02	65.6	1.5	1.2	1.8
		AfterCOVID-19	79	0.02	65.6	1.2	1.0	1.5
	Hospital	12-months before	<5	0.02	2.0	2.0	0.8	7.3
		AfterCOVID-19	13	0.07	2.0	6.6	3.9	12.2

All pain	Community	12-months before	22479	5.1	61.9	363.4	358.7	368.1
		AfterCOVID-19	21564	4.9	62.0	347.6	343.1	352.3
	Hospital	12-months before	989	5.5	1.8	547.1	514.7	582.1
		AfterCOVID-19	897	5.0	1.7	517.4	485.2	552.2
Fatigue	Community	12-months before	1669	0.38	65.3	25.5	24.4	26.8
		AfterCOVID-19	2797	0.64	65.2	42.9	41.3	44.5
	Hospital	12-months before	60	0.33	2.0	30.7	24.0	40.0
		AfterCOVID-19	147	0.81	1.9	76.2	65.0	89.9
Fever	Community	12-months before	580	0.13	65.5	8.9	8.2	9.6
		AfterCOVID-19	259	0.06	65.3	4.0	3.5	4.5
	Hospital	12-months before	30	0.17	2.0	15.3	10.8	22.4
		AfterCOVID-19	35	0.19	1.9	18.3	13.3	26.0
Breathlessness	Community	12-months before	1985	0.45	65.4	30.4	29.1	31.7
		AfterCOVID-19	3048	0.70	65.1	46.7	45.1	48.4
	Hospital	12-months before	258	1.43	1.9	133.8	118.7	151.5
		AfterCOVID-19	505	2.8	1.9	269.9	247.6	294.7
Cough	Community	12-months before	6226	1.4	64.7	96.2	93.9	98.7
		AfterCOVID-19	3114	0.7	64.9	48.0	46.4	49.7
	Hospital	12-months before	387	2.1	1.9	202.6	183.7	224.1
		AfterCOVID-19	254	1.4	1.9	135.2	119.8	153.2
Chest tightness	Community	12-months before	181	0.04	65.6	2.8	2.4	3.2
		AfterCOVID-19	217	0.05	65.6	3.3	2.9	3.8
	Hospital	12-months before	<5	0.02	2.0	2.0	0.8	7.3
		AfterCOVID-19	11	0.06	2.0	5.6	3.2	11.0
Palpitations	Community	12-months before	694	0.16	65.5	10.6	9.8	11.4
		AfterCOVID-19	1079	0.25	65.5	16.5	15.5	17.5
	Hospital	12-months before	25	0.14	2.0	12.8	8.7	19.5
		AfterCOVID-19	74	0.41	2.0	37.9	30.4	48.0
Diarrhea	Community	12-months before	771	0.18	65.5	11.8	11.0	12.6
		AfterCOVID-19	640	0.15	65.5	9.8	9.1	10.6
	Hospital	12-months before	63	0.35	2.0	32.3	25.4	41.7
		AfterCOVID-19	77	0.43	1.9	39.7	31.9	50.0
Nausea	Community	12-months before	294	0.07	65.6	4.5	4.0	5.0

		AfterCOVID-19	412	0.09	65.5	6.3	5.7	6.9
	Hospital	12-months before	11	0.06	2.0	5.6	3.2	11.0
		AfterCOVID-19	54	0.30	1.9	27.7	21.4	36.6
Anorexia	Community	12-months before	202	0.05	65.6	3.1	2.7	3.5
		AfterCOVID-19	202	0.05	65.6	3.1	2.7	3.5
	Hospital	12-months before	10	0.06	2.0	5.1	2.8	10.3
		AfterCOVID-19	20	0.11	2.0	10.2	6.7	16.5
Cognitive impairment	Community	12-months before	150	0.03	65.6	2.3	2.0	2.7
		AfterCOVID-19	180	0.04	65.6	2.7	2.4	3.2
	Hospital	12-months before	26	0.14	2.0	13.3	9.2	20.1
		AfterCOVID-19	43	0.24	2.0	22.0	16.5	30.1
Delirium	Community	12-months before	95	0.02	65.6	1.4	1.2	1.8
		AfterCOVID-19	115	0.03	65.6	1.8	1.5	2.1
	Hospital	12-months before	18	0.10	2.0	9.2	5.9	15.2
		AfterCOVID-19	53	0.29	1.9	27.3	21.0	36.2
Insomnia	Community	12-months before	581	0.13	65.5	8.9	8.2	9.6
		AfterCOVID-19	903	0.21	65.5	13.8	12.9	14.7
	Hospital	12-months before	27	0.15	2.0	13.8	9.6	20.7
		AfterCOVID-19	62	0.34	2.0	31.8	24.9	41.2
Dizziness	Community	12-months before	1438	0.33	65.4	22.0	20.9	23.2
		AfterCOVID-19	1490	0.34	65.4	22.8	21.7	24.0
	Hospital	12-months before	73	0.40	1.9	37.5	29.9	47.5
		AfterCOVID-19	92	0.51	1.9	47.4	38.8	58.5
Paresthesia	Community	12-months before	509	0.12	65.5	7.8	7.1	8.5
		AfterCOVID-19	669	0.15	65.5	10.2	9.5	11.0
	Hospital	12-months before	27	0.15	2.0	13.8	9.6	20.7
		AfterCOVID-19	40	0.22	2.0	20.5	15.1	28.4
Earache	Community	12-months before	966	0.22	65.5	14.8	13.9	15.7
		AfterCOVID-19	948	0.22	65.5	14.5	13.6	15.4
	Hospital	12-months before	37	0.20	2.0	18.9	13.8	26.6
		AfterCOVID-19	33	0.18	2.0	16.9	12.1	24.2
Sore throat	Community	12-months before	2982	0.68	65.1	45.8	44.2	47.5
		AfterCOVID-19	1342	0.31	65.3	20.6	19.5	21.7

	Hospital	12-months before	91	0.50	1.9	46.7	38.2	57.7
		AfterCOVID-19	37	0.20	1.9	19.0	13.9	26.7
Smell/taste loss	Community	12-months before	50	0.01	65.6	0.8	0.6	1.0
		AfterCOVID-19	268	0.06	65.4	4.1	3.6	4.6
	Hospital	12-months before	<5	0.02	2.0	1.5	0.5	7.5
		AfterCOVID-19	6	0.03	1.9	3.1	1.4	8.1
Tinnitus	Community	12-months before	272	0.06	65.6	4.1	3.7	4.7
		AfterCOVID-19	399	0.09	65.5	6.1	5.5	6.7
	Hospital	12-months before	9	0.05	2.0	4.6	2.4	9.8
		AfterCOVID-19	11	0.06	2.0	5.6	3.2	10.9
Skin rash	Community	12-months before	2673	0.61	65.2	41.0	39.5	42.6
		AfterCOVID-19	2284	0.52	65.3	35.0	33.6	36.5
	Hospital	12-months before	81	0.45	1.9	41.6	33.6	52.1
		AfterCOVID-19	77	0.43	1.9	39.5	31.8	49.8

Table S3: Disease event rates (per 100,000 person-weeks).

Outcome	COVID exposure group	Time point	Number of events	% of patients with outcome	Time (person-weeks (PW))	Rate (per 100,000 PW)	Lower CI	Upper CI
**Asthma**	Community	12-months before	4242	0.97	61.8	68.6	66.6	70.7
		AfterCOVID-19	3048	0.70	61.8	49.3	47.6	51.1
	Hospital	12-months before	201	1.11	1.7	114.9	100.2	132.3
		AfterCOVID-19	157	0.87	1.8	89.6	76.8	105.1
**Lung fibrosis**	Community	12-months before	19	0.00	65.6	0.3	0.2	0.5
		AfterCOVID-19	49	0.01	65.6	0.7	0.6	1.0
	Hospital	12-months before	9	0.05	2.0	4.6	2.4	9.8
		AfterCOVID-19	22	0.12	2.0	11.3	7.5	17.7
**Heart failure**	Community	12-months before	182	0.04	65.5	2.8	2.4	3.2
		AfterCOVID-19	200	0.05	65.5	3.1	2.7	3.5
	Hospital	12-months before	39	0.22	1.9	20.3	15.0	28.2
		AfterCOVID-19	125	0.69	1.9	65.6	55.2	78.5
**IHD**	Community	12-months before	439	0.10	65.3	6.7	6.1	7.4
		AfterCOVID-19	380	0.09	65.3	5.8	5.3	6.4
	Hospital	12-months before	47	0.26	1.9	24.6	18.6	33.2
		AfterCOVID-19	116	0.64	1.9	61.0	51.0	73.6
**Hypertension**	Community	12-months before	2884	0.66	63.4	45.5	43.9	47.2
		AfterCOVID-19	2332	0.53	63.7	36.6	35.1	38.1
	Hospital	12-months before	246	1.36	1.8	140.2	124.0	159.3
		AfterCOVID-19	324	1.79	1.8	182.1	163.5	203.5

**Stroke**	Community	12-months before	258	0.06	65.3	3.9	3.5	4.5
		AfterCOVID-19	285	0.07	65.4	4.4	3.9	4.9
	Hospital	12-months before	51	0.28	1.9	26.6	20.3	35.4
		AfterCOVID-19	119	0.66	1.9	62.3	52.2	74.9
**PAD**	Community	12-months before	51	0.01	65.6	0.8	0.6	1.0
		AfterCOVID-19	34	0.01	65.6	0.5	0.4	0.7
	Hospital	12-months before	12	0.07	2.0	6.2	3.6	11.6
		AfterCOVID-19	9	0.05	2.0	4.6	2.5	9.8
**Anaemia**	Community	12-months before	886	0.20	64.9	13.7	12.8	14.6
		AfterCOVID-19	1037	0.24	65.0	16.0	15.0	17.0
	Hospital	12-months before	93	0.51	1.9	49.0	40.1	60.4
		AfterCOVID-19	201	1.11	1.9	107.2	93.5	123.4
**VTE**	Community	12-months before	270	0.06	65.4	4.1	3.7	4.7
		AfterCOVID-19	922	0.21	65.4	14.1	13.2	15.1
	Hospital	12-months before	38	0.21	1.9	19.6	14.4	27.5
		AfterCOVID-19	626	3.47	1.9	335.7	310.3	363.8
**Renal failure**	Community	12-months before	428	0.10	65.3	6.6	6.0	7.2
		AfterCOVID-19	616	0.14	65.3	9.4	8.7	10.2
	Hospital	12-months before	86	0.48	1.9	45.4	36.9	56.5
		AfterCOVID-19	314	1.74	1.9	168.5	150.9	188.7
**GORD**	Community	12-months before	1297	0.30	64.7	20.1	19.0	21.2
		AfterCOVID-19	1423	0.32	64.7	22.0	20.9	23.2
	Hospital	12-months before	53	0.29	1.9	27.6	21.2	36.6
		AfterCOVID-19	76	0.42	1.9	39.6	31.8	50.0

**Liver disease**	Community	12-months before	634	0.14	65.1	9.7	9.0	10.5
		AfterCOVID-19	768	0.18	65.2	11.8	11.0	12.6
	Hospital	12-months before	47	0.26	1.9	24.4	18.5	33.0
		AfterCOVID-19	150	0.83	1.9	78.3	66.8	92.2
**Diabetes**	Community	12-months before	1750	0.40	62.9	27.8	26.5	29.2
		AfterCOVID-19	2173	0.50	63.1	34.4	33.0	35.9
	Hospital	12-months before	190	1.05	1.6	116.4	101.1	134.6
		AfterCOVID-19	457	2.53	1.6	280.0	255.5	307.4
**Adrenal disease**	Community	12-months before	14	0.00	65.6	0.2	0.1	0.4
		AfterCOVID-19	19	0.00	65.6	0.3	0.2	0.5
	Hospital	12-months before	<5	0.01	2.0	1.0	0.2	10.3
		AfterCOVID-19	8	0.04	2.0	4.1	2.1	9.2
**Arthritis**	Community	12-months before	1364	0.31	64.4	21.2	20.1	22.3
		AfterCOVID-19	969	0.22	64.7	15.0	14.1	16.0
	Hospital	12-months before	109	0.60	1.8	59.3	49.3	71.9
		AfterCOVID-19	85	0.47	1.9	45.5	37.0	56.7
**Anxiety**	Community	12-months before	4692	1.07	61.6	76.1	74.0	78.3
		AfterCOVID-19	5160	1.18	61.8	83.5	81.3	85.8
	Hospital	12-months before	129	0.71	1.8	69.8	58.9	83.3
		AfterCOVID-19	240	1.33	1.8	129.9	114.7	147.8
**Depression**	Community	12-months before	3537	0.81	62.7	56.5	54.6	58.4
		AfterCOVID-19	3814	0.87	62.7	60.8	59.0	62.8
	Hospital	12-months before	120	0.66	1.9	64.7	54.3	77.7
		AfterCOVID-19	163	0.90	1.9	87.8	75.5	102.7

Table S4: Prescription event rates (per 100,000 person-weeks).

Outcome	COVID exposure group	Time point	Number of events	% of patients with outcome	Time (person-weeks (PW))	Rate (per 100,000 PW)	Lower CI	Upper CI
**Diuretics**	Community	12-months before	264	0.06	64.6	4.1	3.6	4.6
		AfterCOVID-19	229	0.05	64.5	3.5	3.1	4.0
	Hospital	12-months before	29	0.16	1.8	15.9	11.1	23.4
		AfterCOVID-19	61	0.33	1.8	33.4	26.1	43.4
**Bronchodilators**	Community	12-months before	4472	1.02	60.0	74.6	72.4	76.8
		AfterCOVID-19	3576	0.82	59.6	60.0	58.0	62.0
	Hospital	12-months before	163	0.90	1.6	102.7	88.4	120.1
		AfterCOVID-19	253	1.40	1.6	162.3	143.7	183.9
**ICS**	Community	12-months before	2418	0.55	61.4	39.4	37.9	41.0
		AfterCOVID-19	1958	0.45	60.9	32.1	30.7	33.6
	Hospital	12-months before	115	0.64	1.7	69.1	57.8	83.3
		AfterCOVID-19	125	0.69	1.6	76.7	64.6	91.8
**Paracetamol**	Community	12-months before	822	0.19	64.0	12.8	12.0	13.8
		AfterCOVID-19	756	0.17	64.0	11.8	11.0	12.7
	Hospital	12-months before	94	0.52	1.7	54.8	45.0	67.5
		AfterCOVID-19	321	1.78	1.7	191.5	171.9	214.0
**NSAIDs**	Community	12-months before	5125	1.17	58.5	87.7	85.3	90.1
		AfterCOVID-19	5333	1.22	59.0	90.4	88.0	92.9
	Hospital	12-months before	168	0.93	1.5	110.6	95.3	129.0
		AfterCOVID-19	294	1.63	1.5	192.5	172.0	216.1

**Opiates**	Community	12-months before	4539	1.04	58.9	77.0	74.8	79.3
		AfterCOVID-19	4418	1.01	59.2	74.6	72.4	76.8
	Hospital	12-months before	242	1.34	1.4	168.2	148.6	191.1
		AfterCOVID-19	394	2.18	1.4	279.0	253.1	308.2
**Strong opiates**	Community	12-months before	918	0.21	64.0	14.3	13.4	15.3
		AfterCOVID-19	1093	0.25	64.1	17.0	16.1	18.1
	Hospital	12-months before	75	0.42	1.8	41.8	33.5	52.8
		AfterCOVID-19	231	1.28	1.8	130.8	115.3	149.2
**Weak opiates**	Community	12-months before	4224	0.96	59.9	70.5	68.4	72.7
		AfterCOVID-19	4027	0.92	60.2	66.9	64.9	69.0
	Hospital	12-months before	231	1.28	1.5	150.54	132.63	171.60
		AfterCOVID-19	305	1.69	1.5	200.71	179.71	224.87
**Neuropathic pain medication**	Community	12-months before	2173	0.50	62.1	35.0	33.6	36.5
		AfterCOVID-19	2565	0.59	62.0	41.4	39.8	43.0
	Hospital	12-months before	126	0.70	1.6	76.4	64.4	91.3
		AfterCOVID-19	193	1.07	1.6	117.8	102.6	136.1

Outcome	COVID exposure group	Time point	Number of events	Time (person-weeks (PW))	Rate (per 100,000 PW)	Lower CI	Upper CI
**Total HCU**	Community	12 months before	449290	6559646	6849.3	6829.3	6869.4
		After COVID-19	529975	6559646	8079.3	8057.6	8101.1
	Hospital	12 months before	25429	195981.3	12975.2	12816.2	13135.7
		After COVID-19	43823	195981.3	22360.8	22151.9	22571.2
**Primary care HCU**	Community	12 months before	394882	6559646	6019.9	6001.1	6038.7
		After COVID-19	479970	6559646	7317.0	7296.3	7337.7
	Hospital	12 months before	21138	195981.3	10785.7	10640.8	10932.1
		After COVID-19	37293	195981.3	19028.9	18836.2	19223.0
**Hospital HCU**	Community	12 months before	1711	6559646	26.1	24.9	27.4
		After COVID-19	2050	6559646	31.3	29.9	32.6
	Hospital	12 months before	325	195981.3	165.8	148.3	184.9
		After COVID-19	771	195981.3	393.4	366.1	422.2
**A&E HCU**	Community	12 months before	7266	6559646	110.8	108.2	113.3
		After COVID-19	7932	6559646	120.9	118.3	123.6
	Hospital	12 months before	495	195981.3	252.6	230.8	275.8
		After COVID-19	962	195981.3	490.9	460.3	522.9
**Outpatient HCU**	Community	12 months before	45431	6559646	692.6	686.2	699.0
		After COVID-19	40023	6559646	610.1	604.2	616.2
	Hospital	12 months before	3471	195981.3	1771.1	1712.7	1831.0
		After COVID-19	4797	195981.3	2447.7	2378.9	2518.0

Table S5: Health care utilization (HCU) event rates (per 100,000 person-weeks).

Table S6: Hazard ratios (95% CI) for differences in outcome event rates between patients admitted to hospital with COVID-19 and the same patients 12 months before COVID-19 diagnosis.

Type of outcome	Outcome	Unadjusted HR	Lower 95% CI	Upper 95% CI	P value	Adjusted HR*	Lower 95% CI	Upper 95% CI	P value
**Symptoms**	General pain	1.34	0.82	2.18	0.2492	1.42	0.85	2.38	0.1798
	Chest pain	1.49	1.21	1.85	0.0002	1.39	1.11	1.75	0.0047
	Abdominal pain	1.22	0.99	1.50	0.0638	1.14	0.91	1.43	0.2707
	Headache	0.88	0.67	1.16	0.3654	0.83	0.62	1.12	0.2177
	Joint pain	0.88	0.79	0.99	0.0392	0.85	0.75	0.97	0.0133
	Muscle pain	2.16	1.27	3.67	0.0045	2.07	1.16	3.69	0.0142
	Neuropathic pain	3.25	1.13	9.32	0.028	3.25	1.13	9.32	0.028
	Pain (all)	0.95	0.87	1.03	0.2185	0.91	0.83	1.00	0.0491
	Fatigue	2.48	1.84	3.35	<0.0001	2.52	1.81	3.51	<0.0001
	Fever	1.19	0.73	1.94	0.4801	1.10	0.65	1.86	0.722
	Breathlessness	2.02	1.75	2.33	<0.0001	1.88	1.62	2.19	<0.0001
	Cough	0.67	0.57	0.78	<0.001	0.67	0.57	0.78	<0.001
	Chest tightness	2.75	0.88	8.65	0.083	2.50	0.78	7.99	0.1214

	Palpitations	2.97	1.90	4.64	<0.0001	2.55	1.61	4.05	0.0001
	Diarrhoea	1.23	0.88	1.71	0.2205	1.19	0.85	1.68	0.3087
	Nausea	4.94	2.58	9.44	<0.0001	4.64	2.34	9.21	<0.0001
	Anorexia	2.01	0.94	4.29	0.0722	2.02	0.82	5.02	0.1283
	Cognitive impairment	1.66	1.02	2.68	0.0396	1.66	1.00	2.77	0.0521
	Delirium	2.97	1.74	5.07	0.0001	3.24	1.77	5.94	0.0001
	Insomnia	1.27	0.94	1.71	0.1241	2.17	1.36	3.49	0.0013
	Dizziness	1.27	0.94	1.71	0.1241	1.10	0.79	1.52	0.5828
	Paraesthesia	1.48	0.91	2.42	0.1146	1.35	0.78	2.32	0.279
	Earache	0.89	0.56	1.43	0.6328	0.97	0.58	1.61	0.895
	Sore throat	0.41	0.28	0.60	<0.0001	0.43	0.29	0.65	0.0001
	Smell or taste loss	2.01	0.50	8.05	0.323	2.02	0.50	8.08	0.321
	Tinnitus	1.22	0.53	2.82	0.6379	1.43	0.58	3.54	0.4412
	Skin rash	0.95	0.70	1.30	0.744	1.04	0.74	1.47	0.7996
**Diseases**	Asthma	0.72	0.69	0.75	<0.0001	0.75	0.61	0.93	0.009
	Lung fibrosis	2.58	1.52	4.38	0.0005	2.01	0.90	4.47	0.0877
	Heart failure	1.10	0.90	1.34	0.3571	3.02	2.07	4.42	<0.0001
	IHD	0.87	0.75	0.99	0.0385	2.47	1.72	3.56	<0.0001
	Hypertension	0.80	0.76	0.85	<0.0001	1.22	1.02	1.45	0.0255
	Stroke	1.10	0.93	1.31	0.2508	2.49	1.73	3.59	<0.0001
	PAD	0.67	0.43	1.03	0.0669	0.80	0.32	2.03	0.6403
	Anaemia	1.17	1.07	1.28	0.0007	1.97	1.51	2.55	<0.0001
	VTE	3.42	2.98	3.91	<0.0001	16.21	11.28	23.31	<0.0001
	Renal failure	1.44	1.27	1.63	<0.0001	3.42	2.67	4.38	<0.0001
	GORD	1.10	1.02	1.18	0.0164	1.16	0.80	1.71	0.4341
	Liver disease	1.21	1.09	1.34	0.0004	2.71	1.92	3.83	<0.0001
	Diabetes	1.24	1.16	1.32	<0.0001	2.10	1.76	2.50	<0.0001
	Adrenal disease	1.36	0.68	2.71	0.3859	3.51	0.73	16.90	0.1182
	Arthritis	0.71	0.65	0.77	<0.0001	0.76	0.56	1.03	0.0738
	Anxiety	1.1	1.05	1.14	<0.0001	1.74	1.38	2.19	<0.0001

	Depression	1.08	1.03	1.13	0.0013	1.39	1.08	1.80	0.0107
**Medications**	Diuretics	2.11	1.35	3.28	0.001	1.93	1.19	3.14	0.008
	Bronchodilators	1.58	1.30	1.93	<0.0001	1.44	1.16	1.79	0.0009
	ICS	1.11	0.86	1.43	0.417	1.02	0.77	1.34	0.9112
	Paracetamol	3.50	2.78	4.40	<0.0001	3.68	2.86	4.74	<0.0001
	NSAIDs	1.75	1.45	2.11	<0.0001	1.65	1.34	2.04	<0.0001
	Opiates (all)	1.66	1.42	1.95	0<0.0001	1.57	1.32	1.87	<0.0001
	Weak opiates	1.34	1.13	1.58	0.0009	1.25	1.04	1.51	0.0179
	Strong opiates	3.13	2.42	4.06	<0.0001	2.85	2.16	3.75	<0.0001
	Neuropathic pain medication	1.54	1.23	1.93	0.0001	1.37	1.08	1.74	0.0104
**HCU (IRR)**	Total HCU	1.74	1.70	1.78	<0.0001	1.68	1.64	1.73	<0.0001
	Primary care HCU	1.78	1.74	1.83	<0.0001	1.73	1.68	1.77	<0.0001
	Hospital HCU	2.39	2.02	2.83	<0.0001	2.29	1.94	2.70	<0.0001
	A&E HCU	1.94	1.69	2.22	<0.0001	1.96	1.70	2.25	<0.0001
	Outpatient HCU	1.35	1.29	1.42	<0.0001	1.32	1.25	1.39	<0.0001

Bonferroni significance threshold for symptoms=0.002, for diseases=0.003, for medications=0.005, and for HCU=0.01. *adjusted for age, sex, smoking status, BMI, and CCI.

Table S7: Hazard ratios (95% CI) for differences in outcome event rates between patients not admitted to hospital with CommunityCOVID-19 and the same patients 12 months before COVID-19 diagnosis.

Type of outcome	Outcome	Unadjusted HR	Lower 95% CI	Upper 95% CI	P value	Adjusted HR*	Lower 95% CI	Upper 95% CI	P value
**Symptoms**	General pain	1.21	1.07	1.37	0.0026	1.22	1.06	1.41	0.0054
	Chest pain	1.28	1.21	1.36	<0.0001	1.22	1.14	1.3	<0.0001
	Abdominal pain	1.1	1.06	1.15	<0.0001	1.06	1.01	1.11	0.0286
	Headache	1.09	1.04	1.15	0.0003	1.08	1.02	1.14	0.0086
	Joint pain	0.95	0.93	0.97	0.0001	0.93	0.91	0.96	<0.0001
	Muscle pain	1.83	1.61	2.09	<0.0001	1.89	1.63	2.20	<0.0001
	Neuropathic pain	0.81	0.61	1.09	0.168	0.80	0.60	1.08	0.145
	Pain (all)	0.96	0.94	0.97	<0.0001	0.94	0.92	0.96	<0.0001
	Fatigue	1.68	1.58	1.78	<0.0001	1.64	1.53	1.76	<0.0001
	Fever	0.45	0.39	0.52	<0.0001	0.44	0.37	0.52	<0.0001
	Breathlessness	1.54	1.46	1.63	<0.0001	1.39	1.31	1.47	<0.0001
	Cough	0.50	0.48	0.52	<0.0001	0.49	0.46	0.51	<0.0001
	Chest tightness	1.2	0.99	1.46	0.07	1.17	0.93	1.47	0.179
	Palpitations	1.56	1.42	1.71	<0.0001	1.42	1.27	1.59	<0.0001
	Diarrhoea	0.83	0.75	0.92	0.0004	0.8	0.71	0.91	0.0004
	Nausea	1.4	1.21	1.63	<0.0001	1.24	1.05	1.47	0.0115
	Anorexia	1	0.83	1.21	0.9985	0.98	0.79	1.21	0.8212
	Cognitive impairment	1.2	0.97	1.49	0.0973	1.15	0.9	1.46	0.2626

	Delirium	1.21	0.92	1.59	0.166	1.23	0.92	1.66	0.1661
	Insomnia	1.56	1.4	1.72	<0.0001	1.50	1.33	1.69	<0.0001
	Dizziness	1.04	0.96	1.11	0.3322	1.01	0.93	1.1	0.7317
	Paraesthesia	1.31	1.17	1.47	<0.0001	1.31	1.15	1.49	<0.0001
	Earache	0.98	0.9	1.07	0.6797	1.02	0.92	1.13	0.6966
	Sore throat	0.45	0.42	0.48	<0.0001	0.45	0.42	0.49	<0.0001
	Smell or taste loss	5.37	3.97	7.27	<0.0001	5.28	3.89	7.17	<0.0001
	Tinnitus	1.47	1.26	1.71	<0.0001	1.36	1.14	1.62	0.0007
	Skin rash	0.85	0.81	0.9	<0.0001	0.84	0.79	0.89	<0.0001
**Diseases**	Asthma	0.72	0.69	0.75	<0.0001	0.68	0.65	0.72	<0.0001
	Lung fibrosis	2.58	1.52	4.38	0.0005	2.41	1.37	4.25	0.0023
	Heart failure	1.1	0.9	1.34	0.3571	1.04	0.84	1.29	0.6994
	IHD	0.87	0.75	0.99	0.0385	0.82	0.71	0.95	0.008
	Hypertension	0.8	0.76	0.85	<0.0001	0.75	0.71	0.8	<0.0001
	Stroke	1.1	0.93	1.31	0.2508	1.08	0.9	1.3	0.4128
	PAD	0.67	0.43	1.03	0.0669	0.6	0.38	0.95	0.0309
	Anaemia	1.17	1.07	1.28	0.0007	1.12	1.02	1.24	0.0241
	VTE	3.42	2.98	3.91	<0.0001	3.35	2.87	3.91	<0.0001
	Renal failure	1.44	1.27	1.63	<0.0001	1.33	1.17	1.52	<0.0001
	GORD	1.1	1.02	1.18	0.0164	1.05	0.97	1.15	0.2401
	Liver disease	1.21	1.09	1.34	0.0004	1.14	1.01	1.28	0.0325
	Diabetes	1.24	1.16	1.32	<0.0001	1.13	1.06	1.21	0.0003
	Adrenal disease	1.36	0.68	2.71	0.3859	1.23	0.59	2.56	0.578
	Arthritis	0.71	0.65	0.77	<0.0001	0.71	0.65	0.78	<0.0001
	Anxiety	1.10	1.05	1.14	<0.0001	1.06	1.01	1.11	0.0133
	Depression	1.08	1.03	1.13	0.0013	1.06	1.00	1.12	0.0464
**Medications**	Diuretics	0.87	0.73	1.04	0.115	0.87	0.72	1.05	0.152
	Bronchodilators	0.80	0.77	0.84	<0.0001	0.76	0.73	0.80	<0.0001
	ICS	0.82	0.77	0.87	<0.0001	0.76	0.71	0.82	<0.0001
	Paracetamol	0.92	0.83	1.02	0.1000	0.97	0.86	1.08	0.528

	NSAIDs	1.03	0.99	1.07	0.1132	1.02	0.97	1.06	0.4855
	Opiates (all)	0.97	0.93	1.01	0.1267	0.96	0.92	1.01	0.1148
	Opiates (weak)	0.95	0.91	0.99	0.0181	0.95	0.90	0.99	0.0283
	Opiates (strong)	1.19	1.09	1.30	0.0001	1.18	1.07	1.31	0.001
	Neuropathic pain medication	1.18	1.12	1.25	<0.0001	1.15	1.08	1.23	<0.0001
**HCU (IRR)**	Total HCU	1.19	1.18	1.19	<0.0001	1.15	1.14	1.15	<0.0001
	Primary care HCU	1.22	1.22	1.23	<0.0001	1.18	1.17	1.19	<0.0001
	Hospital HCU	1.21	1.12	1.30	<0.0001	1.16	1.06	1.26	0.0006
	A&E HCU	1.09	1.05	1.13	<0.0001	1.10	1.06	1.15	<0.0001
	Outpatient HCU	0.88	0.86	0.89	<0.0001	0.88	0.86	0.90	<0.0001

Bonferroni significance threshold for symptoms=0.002, for diseases=0.003, for medications=0.005, and for HCU=0.01.*adjusted for age, sex, smoking status, BMI, and CCI.

Table S8: Baseline characteristics for negative control cohort

**Baseline characteristics**	**Negative control cohort**
	**N (%)**
**Sex**	
Male	15,423 (40.1)
Female	23,088 (59.9)
**Age**	
18-30	7,313 (19.0)
31-40	7,106 (18.5)
41-50	6,696 (17.4)
51-60	6,919 (18.0)
61-70	4,597 (11.9)
71-80	3,277 (8.5)
>80	2,603 (6.8)
**BMI (kg/m^2)**	
≤18.5	758 (2.0)
18.5-24.5	8,344 (21.7)
25.0-29.9	9,084 (23.6)
≥ 30.0	10,810 (28.1)
Unknown	9,515 (24.7)
**Smoking status**	
Current smoker	9,736 (25.3)
Ex-smoker	18,728 (48.6)
Never smoked	9,239 (24.0)
Unknown	808 (2.1)
**Baseline diseases (CCI)**	
0	16,716 (43.4)
1-2	14,873 (38.6)
3-4	4,365 (11.3)
5-6	1,745 (4.5)
>6	812 (2.1)
**N**	**38,511**

Table S9: Hazard ratios (95% CI) for differences in outcome event rates between patients in the negative control group and the same patients 12 months before index date.

Type of outcome	Outcome	Unadjusted HR	Lower 95% CI	Upper 95% CI	P value	Adjusted HR*	Lower 95% CI	Upper 95% CI	P value
**Symptoms**	General pain	1.22	0.99	1.51	0.0581	1.31	1.04	1.65	0.0198
	Chest pain	1.18	1.07	1.3	0.0014	1.17	1.05	1.31	0.0041
	Abdominal pain	1.06	0.97	1.15	0.1845	1.04	0.94	1.14	0.4647
	Headache	1.12	1.01	1.24	0.0252	1.15	1.03	1.28	0.0142
	Joint pain	1.02	0.97	1.07	0.5224	1.01	0.96	1.07	0.6466
	Muscle pain	1.71	1.37	2.15	<0.0001	1.7	1.34	2.17	<0.0001
	Neuropathic pain	1	0.59	1.69	0.9989	0.87	0.5	1.53	0.6383
	Pain (all)	0.96	0.92	1.00	00355	0.96	0.92	1.01	0.0889
	Fatigue	1.4	1.24	1.59	<0.0001	1.46	1.28	1.68	<0.0001
	Fever	0.61	0.5	0.76	<0.0001	0.61	0.49	0.77	<0.0001
	Breathlessness	0.88	0.82	0.95	0.0006	0.92	0.85	0.99	0.0339
	Cough	0.48	0.44	0.51	<0.0001	0.49	0.45	0.53	<0.0001
	Chest tightness	1.03	0.73	1.46	0.8476	1.07	0.71	1.61	0.746
	Palpitations	1.27	1.06	1.53	0.0108	1.31	1.08	1.6	0.0066
	Diarrhoea	1.07	0.91	1.26	0.4026	1.09	0.91	1.3	0.3436
	Nausea	1.13	0.89	1.43	0.3223	1.19	0.92	1.53	0.1828
	Anorexia	1.05	0.78	1.41	0.7421	1.12	0.81	1.55	0.4784
	Cognitive impairment	1.15	0.86	1.53	0.343	1.15	0.85	1.57	0.364
	Delirium	2.15	1.49	3.1	<0.0001	2.25	1.51	3.35	0.0001
	Insomnia	1.61	1.34	1.94	<0.0001	1.63	1.33	2	<0.0001
	Dizziness	1.17	1.02	1.35	0.0244	1.24	1.06	1.44	0.0062
	Paraesthesia	1.32	1.04	1.66	0.0199	1.23	0.96	1.57	0.1081
	Earache	1.09	0.92	1.31	0.3228	1.02	0.84	1.24	0.8588
	Sore throat	0.5	0.44	0.58	<0.0001	0.54	0.46	0.63	<0.0001
	Smell or taste loss	0.85	0.52	1.37	0.4931	0.9	0.52	1.55	0.701
	Tinnitus	1.27	0.93	1.73	0.1383	1.05	0.75	1.47	0.774

	Skin rash	0.9	0.8	1.02	0.1012	0.97	0.85	1.12	0.7053
**Diseases**	Asthma	0.88	0.8	0.97	0.009	0.83	0.75	0.92	0.0004
	Lung fibrosis	1	0.54	1.86	0.9973	0.95	0.49	1.84	0.8728
	Heart failure	2.19	1.7	2.82	<0.0001	2.09	1.6	2.73	<0.0001
	IHD	1.14	0.91	1.42	0.2428	1.08	0.86	1.36	0.4891
	Hypertension	1.07	0.95	1.2	0.2468	1.01	0.89	1.14	0.8853
	Stroke	1.04	0.79	1.38	0.7842	0.97	0.72	1.31	0.8412
	PAD	1.32	0.79	2.22	0.295	1.25	0.73	2.13	0.4129
	Anaemia	1.39	1.17	1.66	0.0002	1.29	1.07	1.56	0.0067
	VTE	1.71	1.31	2.25	0.0001	1.51	1.13	2.03	0.0054
	Renal failure	1.41	1.15	1.72	0.0008	1.26	1.02	1.56	0.0314
	GORD	1.35	1.15	1.59	0.0002	1.26	1.05	1.5	0.0109
	Liver disease	1.43	1.16	1.77	0.0009	1.36	1.09	1.7	0.0063
	Diabetes	1.31	1.14	1.5	0.0002	1.23	1.07	1.42	0.0047
	Adrenal disease	1.25	0.49	3.17	0.6382	1.13	0.43	2.91	0.8082
	Arthritis	0.94	0.81	1.09	0.3867	0.93	0.79	1.08	0.3427
	Anxiety	1.17	1.07	1.28	0.0008	1.15	1.04	1.27	0.0071
	Depression	1.24	1.13	1.37	<0.0001	1.23	1.1	1.38	0.0002
**Medications**	Diuretics	0.12	0.1	0.14	<0.0001	0.12	0.1	0.15	<0.0001
	Bronchodilators	0.75	0.69	0.82	<0.0001	0.73	0.66	0.8	<0.0001
	ICS	1.1	1	1.23	0.0619	1.02	0.91	1.14	0.7233
	Paracetamol	0.62	0.53	0.71	<0.0001	0.62	0.53	0.72	<0.0001
	NSAIDs	1.02	0.93	1.11	0.7211	0.99	0.9	1.09	0.8714
	Opiates (all)	1	0.92	1.08	0.9405	1.02	0.93	1.12	0.6541
	Weak opiates	0.97	0.89	1.06	0.5006	0.98	0.9	1.08	0.7401
	Strong opiates	1.24	1.07	1.43	0.0042	1.27	1.09	1.49	0.0026
	Neuropathic pain medication	1.11	0.99	1.24	0.0731	1.11	0.98	1.25	0.1169
**HCU (IRR)**	Total HCU	1.17	1.16	1.19	<0.0001	1.16	1.15	1.18	<0.0001
	Primary care HCU	1.19	1.18	1.21	<0.0001	1.18	1.16	1.2	<0.0001
	Hospital HCU	1.32	1.17	1.49	<0.0001	1.29	1.13	1.47	0.0002

	A&E HCU	1.19	1.1	1.27	<0.0001	1.21	1.12	1.32	<0.0001
	Outpatient HCU	0.97	0.94	1.01	0.102	0.97	0.94	1.01	0.1013

Bonferroni significance threshold for symptoms=0.002, for diseases=0.003, for medications=0.005, and for HCU=0.01. *adjusted for age, sex, smoking status, BMI, and CCI.

Table S10: Hazard ratios (95% CI) for differences in outcome event rates between patients with influenza and the same patients 12 months before influenza diagnosis.

Type of outcome	Outcome	Unadjusted HR	Lower 95% CI	Upper 95% CI	P value	Adjusted HR*	Lower 95% CI	Upper 95% CI	P value
**Symptoms**	General pain	0.92	0.72	1.18	0.5183	0.92	0.7	1.2	0.5305
	Chest pain	1.08	0.93	1.25	0.3008	1.06	0.91	1.25	0.4418
	Abdominal pain	1.1	0.98	1.24	0.092	1.09	0.96	1.24	0.1666
	Headache	0.78	0.68	0.9	0.0005	0.81	0.69	0.94	0.0057
	Joint pain	0.98	0.91	1.04	0.453	0.96	0.9	1.04	0.3378
	Muscle pain	1.1	0.79	1.53	0.5688	1.1	0.76	1.6	0.6012
	Neuropathic pain	1	0.58	1.72	0.9995	1	0.54	1.86	0.999
	Pain (all)	0.94	0.89	0.98	0.0095	0.93	0.88	1.07	0.5557
	Fatigue	1.11	0.96	1.29	0.1703	0.89	0.69	1.15	0.3832
	Fever	0.96	0.7	1.32	0.8071	1.49	0.98	2.27	0.0631
	Breathlessness	1.22	1.08	1.39	0.0013	0.64	0.35	1.19	0.1585
	Cough	1.01	0.92	1.1	0.9046	1.15	0.87	1.52	0.3251
	Chest tightness	1.11	0.59	2.1	0.7439	1.12	0.82	1.53	0.4734
	Palpitations	1.11	0.86	1.43	0.4101	1.37	0.67	2.82	0.3854
	Diarrhoea	0.88	0.7	1.1	0.2491	1.07	0.9	1.27	0.4502
	Nausea	1.07	0.71	1.6	0.7501	0.88	0.5	1.55	0.6693
	Anorexia	0.65	0.36	1.15	0.1385	0.96	0.67	1.4	0.8493
	Cognitive impairment	1.66	1.13	2.44	0.0104	1.17	1.03	1.33	0.0184
	Delirium	1.21	0.63	2.35	0.5636	0.98	0.69	1.4	0.9161
	Insomnia	1.23	0.92	1.63	0.1649	1.43	0.88	2.33	0.1443
	Dizziness	1.14	0.96	1.35	0.1408	0.97	0.88	1.07	0.5557
	Paraesthesia	1	0.71	1.4	0.9965	1.24	0.9	1.7	0.1869
	Earache	1.09	0.82	1.46	0.54	0.74	0.62	0.87	0.0003
	Sore throat	0.77	0.66	0.89	0.0004	1.08	0.89	1.31	0.4268
	Smell or taste loss	4	0.45	35.79	0.215	0.95	0.8	1.12	0.5286
	Tinnitus	1.41	0.87	2.29	0.1674	1.14	0.56	2.35	0.713

	Skin rash	0.95	0.82	1.1	0.4658	4	0.45	35.87	0.2157
**Diseases**	Asthma	1.08	0.92	1.26	0.3428	1.02	0.86	1.2	0.8551
	Lung fibrosis	1.17	0.39	3.47	0.7813	1.26	0.34	4.7	0.7336
	Heart failure	1.57	0.98	2.53	0.0605	1.73	1.04	2.86	0.0335
	IHD	1.08	0.8	1.46	0.5973	1.05	0.77	1.42	0.7614
	Hypertension	0.93	0.81	1.07	0.3206	0.86	0.74	1	0.0523
	Stroke	1.1	0.75	1.61	0.6251	0.96	0.64	1.45	0.845
	PAD	1	0.5	2	0.9993	1.16	0.55	2.43	0.6969
	Anaemia	1.61	1.27	2.04	0.0001	1.63	1.25	2.11	0.0003
	VTE	1.57	0.98	2.52	0.0612	1.59	0.95	2.65	0.0764
	Renal failure	0.93	0.68	1.27	0.6498	0.88	0.63	1.21	0.4271
	GORD	1.09	0.87	1.37	0.4501	1.03	0.8	1.32	0.8359
	Liver disease	1.62	1.21	2.16	0.0011	1.53	1.11	2.11	0.01
	Diabetes	1.05	0.86	1.29	0.6242	0.97	0.79	1.19	0.7669
	Adrenal disease	2	0.5	8	0.3268	2	0.5	8	0.3267
	Arthritis	0.84	0.7	1.02	0.0746	0.87	0.71	1.07	0.186
	Anxiety	1.01	0.88	1.16	0.8907	0.96	0.81	1.13	0.6168
	Depression	1.07	0.92	1.26	0.3664	1.06	0.88	1.27	0.5355
**Medications**	Diuretics	0.06	0.05	0.08	<0.0001	0.07	0.05	0.1	<0.0001
	Bronchodilators	0.99	0.85	1.16	0.8877	0.93	0.78	1.11	0.4276
	ICS	1.13	0.91	1.4	0.2608	1.12	0.89	1.41	0.3393
	Paracetamol	0.87	0.69	1.1	0.242	0.83	0.64	1.06	0.1352
	NSAIDs	0.89	0.79	1	0.0467	0.84	0.74	0.96	0.0124
	Opiates (all)	1.01	0.9	1.14	0.8112	1.02	0.9	1.16	0.733
	Weak opiates	1.03	0.91	1.16	0.6654	1.02	0.9	1.17	0.7396
	Strong opiates	0.86	0.69	1.08	0.1979	0.91	0.71	1.16	0.4307
	Neuropathic pain medication	1.08	0.92	1.28	0.3504	1.03	0.86	1.24	0.7544
**HCU (IRR)**	Total HCU	1.05	1.03	1.07	<0.0001	1.03	1.01	1.05	0.0037
	Primary care HCU	1.04	1.02	1.06	<0.0001	1.02	1	1.04	0.0289
	Hospital HCU	0.98	0.82	1.16	0.7903	1	0.82	1.21	0.9726

	A&E HCU	1.03	0.92	1.15	0.5863	1.04	0.92	1.17	0.5549
	Outpatient HCU	1.11	1.07	1.16	<0.0001	1.09	1.04	1.14	0.0003

Bonferroni significance threshold for symptoms=0.002, for diseases=0.003, for medications=0.005, and for HCU=0.01. *adjusted for age, sex, smoking status, BMI, and CCI.

Table S11: Event rates (per 100,000 person-weeks) for patients not admitted to hospital for Community COVID-19 in the period from COVID-19 diagnosis to vaccination and from vaccination to end of follow-up.

**Outcome**	**Time point**	**Number of events**	**Time (person-weeks (PW))**	**Rate (per 100,000 PW)**	**Lower CI**	**Upper CI**
General pain	Beforevaccine	392	291670.8	134.4	121.4	148.4
	Aftervaccine	138	165268.1	83.5	70.2	98.7
Chest pain	Before vaccine	2264	291670.8	776.3	744.6	809.0
	After vaccine	363	165268.1	219.6	197.6	243.4
Abdominal pain	Before vaccine	2909	291670.8	997.5	961.5	1034.4
	After vaccine	613	165268.1	370.9	342.1	401.5
Headache	Before vaccine	2178	291670.8	746.8	715.8	778.8
	After vaccine	616	165268.1	372.7	343.9	403.4
Joint pain	Before vaccine	9492	291670.8	3254.7	3189.5	3320.8
	After vaccine	2271	165268.1	1374.1	1318.2	1431.8
Muscle pain	Before vaccine	495	291670.8	169.7	155.1	185.4
	After vaccine	175	165268.1	105.9	90.8	122.8
Neuropathic pain	Before vaccine	67	291670.8	23.0	17.8	29.2
	After vaccine	23	165268.1	13.9	8.8	20.9
Pain (all)	Before vaccine	18956	291670.8	6499.8	6407.6	6593.0
	After vaccine	4462	165268.1	2699.89	2621.2	2780.3
Fatigue	Before vaccine	1818	291670.8	623.4	595.0	652.7
	After vaccine	330	165268.1	199.7	178.7	222.4
Fever	Before vaccine	143	291670.8	49.0	41.3	57.8
	After vaccine	23	165268.1	13.9	8.8	20.9
Breathlessness	Before vaccine	2913	291670.8	998.8	962.9	1035.8
	After vaccine	582	165268.1	352.2	324.1	382.0
Cough	Before vaccine	2750	291670.8	942.9	908.0	978.9
	After vaccine	437	165268.1	264.4	240.2	290.4
Chest tightness	Before vaccine	157	291670.8	53.8	45.7	62.9
	After vaccine	14	165268.1	8.5	4.6	14.2
Palpitations	Before vaccine	801	291670.8	274.7	256.0	294.4
	After vaccine	196	165268.1	118.6	102.6	136.4
Diarrhea	Before vaccine	444	291670.8	152.2	138.4	167.1

	After vaccine	84	165268.1	50.8	40.5	62.9
Nausea	Before vaccine	175	291670.8	60.0	51.4	69.6
	After vaccine	40	165268.1	24.2	17.3	33.0
Anorexia	Before vaccine	112	291670.8	38.4	31.6	46.2
	After vaccine	17	165268.1	2.5	6.0	16.5
Cognitive impairment	Before vaccine	176	291670.8	60.4	51.8	70.0
	After vaccine	57	165268.1	34.5	26.1	44.7
Delirium	Before vaccine	116	291670.8	39.8	32.9	47.7
	After vaccine	26	165268.1	15.7	10.3	23.1
Insomnia	Before vaccine	573	291670.8	196.5	180.7	213.2
	After vaccine	94	165268.1	56.9	46.0	69.6
Dizziness	Before vaccine	1195	291670.8	409.8	386.8	433.7
	After vaccine	265	165268.1	160.4	141.6	180.9
Paresthesia	Before vaccine	473	291670.8	162.2	147.9	177.5
	After vaccine	97	165268.1	58.7	47.6	71.6
Earache	Before vaccine	579	291670.8	198.5	182.7	215.4
	After vaccine	137	165268.1	82.9	69.6	98
Sore throat	Before vaccine	647	291670.8	221.9	205.1	239.6
	After vaccine	149	165268.1	90.2	76.3	105.9
Smell/taste loss	Before vaccine	183	291670.8	62.8	54.0	72.5
	After vaccine	21	165268.1	2.8	7.8	19.4
Tinnitus	Before vaccine	294	291670.8	100.8	89.6	113.0
	After vaccine	69	165268.1	41.8	32.5	52.8
Skin rash	Before vaccine	1220	291670.8	418.3	395.2	442.5
	After vaccine	242	165268.1	146.4	128.6	166.1

Table S12: Event rates (per 100,000 person-weeks) for patients not admitted to hospital with Community COVID-19 in the period from COVID-19 diagnosis to vaccination and from vaccination to end of follow-up.

**Outcome**	**Time point**	**Number of events**	**Time (person-weeks (PW))**	**Rate (per 100,000 PW)**	**Lower CI**	**Upper CI**
Asthma	Before vaccine	8483	291670.8	2908.42	2846.85	2970.98
	After vaccine	2939	165268.1	1778.32	1714.6	1843.8
Lung fibrosis	Before vaccine	26	291670.8	8.91	5.82	13.06

	After vaccine	29	165268.1	17.55	11.75	25.2
Heart failure	Before vaccine	166	291670.8	56.91	48.58	66.26
	After vaccine	92	165268.1	55.67	44.88	68.27
IHD	Before vaccine	363	291670.8	124.46	111.98	137.94
	After vaccine	119	165268.1	72	59.65	86.16
Hypertension	Before vaccine	978	291670.8	335.31	314.62	357
	After vaccine	605	165268.1	366.07	337.48	396.44
Stroke	Before vaccine	93	291670.8	31.89	25.74	39.06
	After vaccine	55	165268.1	33.28	25.07	43.32
PAD	Before vaccine	6	291670.8	2.06	0.75	4.48
	After vaccine	14	165268.1	8.47	4.63	14.21
Anaemia	Before vaccine	318	291670.8	109.03	97.37	121.69
	After vaccine	177	165268.1	107.1	91.9	124.09
VTE	Before vaccine	250	291670.8	85.71	75.42	97.02
	After vaccine	81	165268.1	49.01	38.92	60.92
Renal failure	Before vaccine	179	291670.8	61.37	52.71	71.05
	After vaccine	101	165268.1	61.11	49.78	74.26
GORD	Before vaccine	343	291670.8	117.6	105.48	130.73
	After vaccine	128	165268.1	77.45	64.61	92.09
Liver disease	Before vaccine	243	291670.8	83.3	73.17	94.47
	After vaccine	140	165268.1	84.71	71.26	99.96
Diabetes	Before vaccine	2155	291670.8	738.85	707.98	770.71
	After vaccine	1410	165268.1	853.16	809.2	898.88
Adrenal disease	Before vaccine	9	291670.8	3.09	1.41	5.86
	After vaccine	<5	165268.1	1.82	0.37	5.3
Thyroid disease	Before vaccine	286	291670.8	98.06	87.02	110.1
	After vaccine	159	165268.1	96.21	81.83	112.38
Arthritis	Before vaccine	531	291670.8	182.05	166.9	198.22
	After vaccine	393	165268.1	237.8	214.86	262.51
Anxiety	Before vaccine	1484	291670.8	508.79	483.23	535.35
	After vaccine	779	165268.1	471.36	438.83	505.65
Depression	Before vaccine	1115	291670.8	382.28	360.17	405.39
	After vaccine	646	165268.1	390.88	361.31	422.22

Table S13: Event rates (per 100,000 person-weeks) for patients not admitted to hospital for Community COVID-19 in the period from COVID-19 diagnosis to vaccination and from vaccination to end of follow-up.

**Outcome**	**Time point**	**Number of events**	**Time (person-weeks (PW))**	**Rate (per 100,000 PW)**	**Lower CI**	**Upper CI**
Diuretics	Before vaccine	1792	291670.8	614.39	586.27	643.51

	After vaccine	1244	165268.1	752.72	711.46	795.74
Bronchodilators	Before vaccine	6833	291670.8	2342.71	2287.49	2398.93
	After vaccine	3376	165268.1	2042.74	1974.41	2112.83
ICS	Before vaccine	5891	291670.8	2019.74	1968.49	2071.99
	After vaccine	3203	165268.1	1938.06	1871.52	2006.37
Paracetamol	Before vaccine	2823	291670.8	967.87	932.49	1004.25
	After vaccine	1862	165268.1	1126.65	1076.06	1179.02
NSAIDS	Before vaccine	6757	291670.8	2316.65	2261.74	2372.56
	After vaccine	3522	165268.1	2131.08	2061.28	2202.65
Opiates (all)	Before vaccine	10218	291670.8	3503.26	3435.66	3571.86
	After vaccine	5369	165268.1	3248.66	3162.34	3336.74
Strong opiates	Before vaccine	3821	291670.8	1310.04	1268.83	1352.25
	After vaccine	2343	165268.1	1417.7	1360.87	1476.29
Weak opiates	Before vaccine	6397	291670.8	2193.23	2139.81	2247.64
	After vaccine	3026	165268.1	1830.96	1766.3	1897.39
Neuropathic pain medication	Before vaccine	8344	291670.8	2860.76	2799.7	2922.81
	After vaccine	4877	165268.1	2950.96	2868.72	3034.97

Table S14: Event rates (per 100,000 person-weeks) for patients not admitted to hospital for Community COVID-19 in the period from COVID-19 diagnosis to vaccination and from vaccination to end of follow-up.

**HCU**	**Time point**	**Number of events**	**Time (person-weeks (PW))**	**Rate (per 100,000 PW)**	**Lower CI**	**Upper CI**
Total HCU	Before vaccine	74368	291670.8	25497.2	25314.3	25681.2

	After vaccine	22616	165268.1	13684.4	13506.7	13864.0
Primary care HCU	Before vaccine	67309	291670.8	23077.0	22903.0	23252.1
	After vaccine	20044	165268.1	12128.2	11960.8	12297.3
Hospital HCU	Before vaccine	474	291670.8	162.5	148.2	177.8
	After vaccine	101	165268.1	61.1	49.8	74.3
A&E HCU	Before vaccine	1189	291670.8	407.7	384.8	431.5
	After vaccine	358	165268.1	216.6	194.8	240.3
Outpatient HCU	Before vaccine	5396	291670.8	1850.0	1801.0	1900.1
	After vaccine	2113	165268.1	1278.5	1224.6	1334.2

Table S15: IRR (95% CI) for differences in outcome event rates between symptomatic, vaccinated patients who were not admitted to hospital for Community COVID-19 and the same patients after vaccination.

**Type of outcome**	**Outcome**	**Unadjusted IRR**	**Lower 95% CI**	**Upper 95% CI**	**P value**	**Adjusted* IRR**	**Lower 95% CI**	**Upper 95% CI**	**P value**
**Symptoms**	General pain	0.57	0.45	0.73	<0.0001	0.64	0.46	0.89	0.0079
	Chest pain	0.26	0.22	0.30	<0.0001	0.40	0.33	0.48	<0.0001
	Abdominal pain	0.36	0.32	0.40	<0.0001	0.44	0.38	0.52	<0.0001
	Headache	0.47	0.42	0.54	<0.0001	0.64	0.54	0.77	<0.0001

	Joint pain	0.40	0.38	0.43	<0.0001	0.55	0.51	0.60	<0.0001
	Muscle pain	0.56	0.46	0.69	<0.0001	0.71	0.53	0.95	0.0198
	Neuropathic pain	0.54	0.27	1.05	0.071	0.71	0.36	1.40	0.3231
	Pain (all)	0.40	0.38	0.42	<0.0001	0.54	0.52	0.58	<0.0001
	Fatigue	0.31	0.26	0.35	<0.0001	0.42	0.35	0.50	<0.0001
	Fever	0.27	0.16	0.44	<0.0001	0.47	0.27	0.82	0.0071
	Breathlessness	0.32	0.29	0.36	<0.0001	0.48	0.42	0.56	<0.0001
	Cough	0.25	0.22	0.29	<0.0001	0.40	0.34	0.47	<0.0001
	Chest tightness	0.15	0.07	0.34	<0.0001	0.15	0.07	0.36	<0.0001
	Palpitations	0.40	0.33	0.48	<0.0001	0.63	0.48	0.83	0.0009
	Diarrhoea	0.31	0.23	0.41	<0.0001	0.45	0.31	0.66	<0.0001
	Nausea	0.39	0.27	0.56	<0.0001	0.43	0.29	0.66	<0.0001
	Anorexia	0.25	0.12	0.52	0.0002	0.32	0.16	0.64	0.0013
	Cognitive impairment	0.45	0.32	0.65	<0.0001	0.81	0.47	1.39	0.4463
	Delirium	0.33	0.20	0.54	<0.0001	0.44	0.24	0.83	0.0116
	Insomnia	0.27	0.20	0.37	<0.0001	0.44	0.30	0.63	<0.0001
	Dizziness	0.36	0.30	0.43	<0.0001	0.49	0.39	0.62	<0.0001
	Paraesthesia	0.35	0.27	0.46	<0.0001	0.48	0.34	0.66	<0.0001
	Earache	0.39	0.31	0.49	<0.0001	0.52	0.37	0.71	0.0001
	Sore throat	0.38	0.31	0.48	<0.0001	0.55	0.42	0.73	<0.0001
	Smell or taste loss	0.19	0.11	0.32	<0.0001	0.32	0.17	0.58	0.0002
	Tinnitus	0.39	0.28	0.56	<0.0001	0.39	0.25	0.59	<0.0001
	Skin rash	0.33	0.29	0.39	<0.0001	0.40	0.32	0.50	<0.0001
**Diseases**	Asthma	0.52	0.46	0.59	<0.0001	0.63	0.49	0.82	0.0006
	Lung fibrosis	1.46	0.83	2.57	0.1936	0.90	0.42	1.94	0.7964
	Heart failure	0.84	0.6	1.17	0.2972	0.88	0.44	1.75	0.7171
	IHD	0.52	0.4	0.66	<0.0001	0.41	0.27	0.63	0.0001
	Hypertension	1.02	0.89	1.16	0.7953	1.15	0.95	1.39	0.1479
	Stroke	0.92	0.61	1.37	0.6747	0.86	0.45	1.65	0.6558
	PAD	4.19	1.76	9.98	0.0012	2.01	0.49	8.24	0.3323
	Anaemia	0.87	0.69	1.11	0.2664	0.91	0.63	1.31	0.6007

	VTE	0.47	0.34	0.64	<0.0001	0.86	0.57	1.29	0.455
	Renal failure	0.94	0.72	1.23	0.6538	0.66	0.43	1.00	0.0515
	GORD	0.65	0.51	0.81	0.0002	0.68	0.51	0.89	0.0058
	Liver disease	0.95	0.73	1.25	0.7332	1.16	0.84	1.60	0.88
	Diabetes	0.99	0.89	1.09	0.8076	1.15	0.97	1.37	0.0981
	Adrenal disease	0.55	0.11	2.91	0.4838	1.04	0.15	7.45	0.9665
	Arthritis	1.22	1.04	1.45	0.018	1.18	0.91	1.54	0.2107
	Anxiety	0.89	0.79	1.00	0.0569	1.00	0.84	1.20	0.9906
	Depression	1.01	0.89	1.14	0.9271	0.9	0.74	1.09	0.2824
**Medications**	Diuretics	0.95	0.89	1.01	0.1191	0.72	0.66	0.78	<0.0001
	Bronchodilators	0.75	0.72	0.77	<0.0001	0.80	0.74	0.86	<0.0001
	ICS	0.86	0.83	0.89	<0.0001	0.89	0.81	0.99	0.0246
	Paracetamol	0.91	0.87	0.96	0.0003	0.85	0.73	1.00	0.0454
	NSAIDs	0.81	0.78	0.84	<0.0001	0.82	0.75	0.88	<0.0001
	Opiates (all)	0.8	0.78	0.83	<0.0001	0.77	0.71	0.84	<0.0001
	Weak opiates	0.75	0.72	0.78	0	0.71	0.65	0.78	<0.0001
	Strong opiates	0.9	0.86	0.95	0.0001	0.89	0.77	1.03	0.1292
	Neuropathic pain medication	0.89	0.86	0.92	<0.0001	0.89	0.81	0.99	0.0246
**HCU**	All HCU	0.5	0.49	0.51	<0.0001	0.50	0.48	0.51	<0.0001
	Primary care HCU	0.49	0.48	0.50	<0.0001	0.50	0.48	0.51	<0.0001
	A&E HCU	0.51	0.45	0.58	<0.0001	0.59	0.50	0.70	<0.0001
	Hospital HCU	0.36	0.29	0.45	<0.0001	0.29	0.21	0.38	<0.0001
	Outpatient HCU	0.66	0.62	0.70	<0.0001	0.57	0.52	0.62	<0.0001

Bonferroni significance threshold for symptoms=0.002, for diseases=0.003, for medications=0.005, and for HCU=0.01. *adjusted for age, sex, smoking status, BMI, and CCI with the exception of nausea, VTE and liver disease which were adjusted for age, sex, smoking status and CCI due to model convergence.

Table S16: Symptom event rates (per 100,000 person-weeks) for 2-week washout sensitivity analysis.

Outcome	COVID exposure group	Time point	Number of events	Time (person-weeks (PW))	Rate (per 100,000 PW)	Lower CI	Upper CI
**General pain**	Community	12-months before	532	65.5	8.1	7.5	8.8
		After COVID-19	627	65.5	9.6	8.9	10.4

	Hospital	12-months before	38	2.0	19.4	14.3	27.2
		After COVID-19	45	2.0	23.0	17.3	31.3
**Chest pain**	Community	12-months before	2510	65.2	38.5	37.0	40.0
		After COVID-19	3427	65.1	52.6	50.9	54.4
	Hospital	12-months before	174	1.9	89.9	77.7	104.6
		After COVID-19	290	1.9	151.9	135.6	170.8
**Abdominal pain**	Community	12-months before	4499	64.9	69.3	67.3	71.4
		After COVID-19	4711	64.9	72.6	70.5	74.7
	Hospital	12-months before	189	1.9	97.9	85.1	113.2
		After COVID-19	236	1.9	123.8	109.2	141.0
**Headache**	Community	12-months before	3621	65.0	55.7	53.9	57.5
		After COVID-19	4170	64.8	64.3	62.4	66.3
	Hospital	12-months before	147	1.9	75.7	64.6	89.3
		After COVID-19	135	1.9	70.6	59.8	83.9
**Joint pain**	Community	12-months before	14197	63.4	223.9	220.3	227.7
		After COVID-19	12742	63.7	200.1	196.7	203.7
	Hospital	12-months before	723	1.9	387.9	360.9	417.5
		After COVID-19	588	1.8	318.0	293.6	344.9
**Muscle pain**	Community	12-months before	420	65.5	6.4	5.8	7.1
		After COVID-19	792	65.5	12.1	11.3	13.0
	Hospital	12-months before	32	2.0	16.4	11.7	23.6
		After COVID-19	67	1.9	34.4	27.2	44.1
**Neuropathic pain**	Community	12-months before	117	65.6	1.8	1.5	2.1
		After COVID-19	95	65.6	1.4	1.2	1.8
	Hospital	12-months before	6	2.0	3.1	1.4	8.1
		After COVID-19	13	2.0	6.6	3.9	12.2
**Pain (all)**	Community	12-months before	27122	61.3	442.5	427.3	447.9
		After COVID-19	25370	61.5	412.2	407.2	417.3
	Hospital	12-months before	1263	1.8	708.7	671.0	749.0
		After COVID-19	1162	1.7	679.8	642.2	720.1
**Fatigue**	Community	12-months before	2026	65.3	31.0	29.7	32.4
		After COVID-19	3542	65.1	54.4	52.6	56.2
	Hospital	12-months before	81	1.9	41.5	33.6	52.0
		After COVID-19	212	1.9	110.2	96.5	126.4

**Fever**	Community	12-months before	679	65.5	10.4	9.6	11.2
		After COVID-19	445	65.3	6.8	6.2	7.5
	Hospital	12-months before	34	2.0	17.4	12.5	24.8
		After COVID-19	57	1.9	29.8	23.1	39.1
**Breathlessness**	Community	12-months before	1985	65.4	30.4	29.1	31.7
		After COVID-19	4267	65.0	65.7	63.7	67.7
	Hospital	12-months before	258	1.9	133.8	118.7	151.5
		After COVID-19	673	1.8	368.4	341.8	397.7
**Cough**	Community	12-months before	7557	64.6	117.1	114.5	119.7
		After COVID-19	4509	64.6	69.8	67.8	71.9
	Hospital	12-months before	493	1.9	259.3	237.7	283.5
		After COVID-19	335	1.8	182.2	163.8	203.1
**Chest tightness**	Community	12-months before	211	65.6	3.2	2.8	3.7
		After COVID-19	300	65.6	4.6	4.1	5.1
	Hospital	12-months before	6	2.0	3.1	1.4	8.1
		After COVID-19	14	2.0	7.2	4.3	12.8
**Palpitations**	Community	12-months before	833	65.5	12.7	11.9	13.6
		After COVID-19	1291	65.4	19.7	18.7	20.8
	Hospital	12-months before	41	2.0	21.0	15.6	28.9
		After COVID-19	87	1.9	44.6	36.3	55.5
**Diarrhea**	Community	12-months before	927	65.5	14.2	13.3	15.1
		After COVID-19	828	65.5	12.6	11.8	13.5
	Hospital	12-months before	75	1.9	38.5	30.8	48.6
		After COVID-19	123	1.9	63.5	53.4	76.1
**Nausea**	Community	12-months before	364	65.5	5.6	5.0	6.2
		After COVID-19	494	65.5	7.5	6.9	8.2
	Hospital	12-months before	17	2.0	8.7	5.5	14.6
		After COVID-19	65	1.9	33.4	26.3	43.0
**Anorexia**	Community	12-months before	259	65.6	4.0	3.5	4.5
		After COVID-19	272	65.5	4.1	3.7	4.7
	Hospital	12-months before	13	2.0	6.6	3.9	12.2
		After COVID-19	35	2.0	17.9	13.0	25.5
**Cognitive impairment**	Community	12-months before	206	65.6	3.1	2.7	3.6
		After COVID-19	223	65.6	3.4	3.0	3.9

	Hospital	12-months before	34	2.0	17.4	12.5	24.8
		After COVID-19	60	2.0	30.7	24.0	40.0
**Delirium**	Community	12-months before	124	65.6	1.9	1.6	2.3
		After COVID-19	152	65.6	2.3	2.0	2.7
	Hospital	12-months before	23	2.0	11.8	7.9	18.3
		After COVID-19	83	1.9	42.8	34.7	53.5
**Insomnia**	Community	12-months before	700	65.5	10.7	9.9	11.5
		After COVID-19	1121	65.5	17.1	16.2	18.2
	Hospital	12-months before	40	2.0	20.5	15.1	28.4
		After COVID-19	91	1.9	46.7	38.2	57.7
**Dizziness**	Community	12-months before	1762	65.3	27.0	25.7	28.3
		After COVID-19	1793	65.4	27.4	26.2	28.7
	Hospital	12-months before	99	1.9	50.9	41.9	62.3
		After COVID-19	122	1.9	63.0	52.9	75.6
**Paresthesia**	Community	12-months before	630	65.5	9.6	8.9	10.4
		After COVID-19	758	65.5	11.6	10.8	12.4
	Hospital	12-months before	34	2.0	17.4	12.5	24.8
		After COVID-19	54	2.0	27.6	21.3	36.5
**Earache**	Community	12-months before	1139	65.4	17.4	16.4	18.5
		After COVID-19	1194	65.4	18.3	17.3	19.3
	Hospital	12-months before	43	2.0	22.0	16.5	30.1
		After COVID-19	40	2.0	20.5	15.1	28.4
**Sore throat**	Community	12-months before	3541	65.1	54.4	52.7	56.2
		After COVID-19	1646	65.2	25.2	24.1	26.5
	Hospital	12-months before	108	1.9	55.4	46.1	67.3
		After COVID-19	55	1.9	28.3	21.9	37.3
**Smell/taste loss**	Community	12-months before	52	65.6	0.8	0.6	1.1
		After COVID-19	525	65.4	8.0	7.4	8.8
	Hospital	12-months before	<5	2.0	1.5	0.5	7.5
		After COVID-19	28	1.9	14.4	10.1	21.4
**Tinnitus**	Community	12-months before	341	65.5	5.2	4.7	5.8
		After COVID-19	467	65.5	7.1	6.5	7.8
	Hospital	12-months before	18	2.0	9.2	5.9	15.2
		After COVID-19	14	2.0	7.1	4.3	12.8

**Skin rash**	Community	12-months before	3236	65.1	49.7	48.0	51.5
		After COVID-19	2649	65.2	40.6	39.1	42.2
	Hospital	12-months before	105	1.9	54.0	44.7	65.7
		After COVID-19	96	1.9	49.3	40.5	60.6

Table S17: Prescription event rates (per 100,000 person-weeks) for 2-week washout sensitivity analysis.

Outcome	COVID exposure group	Time point	Number of events	Time (person-weeks (PW))	Rate (per 100,000 PW)	Lower CI	Upper CI
**Diuretics**	Community	12-months before	6467	65.1	99.3	96.9	101.7
		After COVID-19	243	64.6	3.8	3.3	4.3
	Hospital	12-months before	864	1.9	454.3	424.9	486.3
		After COVID-19	73	1.8	40.0	32.0	50.7
**Bronchodilators**	Community	12-months before	5031	59.9	84.0	81.7	86.3
		After COVID-19	4888	59.5	82.2	79.9	84.5
	Hospital	12-months before	192	1.6	121.2	105.5	140.0
		After COVID-19	364	1.5	235.1	212.3	261.0
**ICS**	Community	12-months before	2653	61.3	43.2	41.6	44.9
		After COVID-19	2380	60.9	39.1	37.6	40.7
	Hospital	12-months before	133	1.7	80.0	67.7	95.2
		After COVID-19	158	1.6	97.1	83.3	113.9
**Paracetamol**	Community	12-months before	941	64.0	14.7	13.8	15.7
		After COVID-19	969	63.9	15.2	14.2	16.2
	Hospital	12-months before	113	1.7	65.9	55.0	79.7
		After COVID-19	430	1.7	257.9	234.8	283.9
**NSAIDs**	Community	12-months before	6102	58.3	104.6	102.0	107.3
		After COVID-19	6141	58.9	104.3	101.7	106.9
	Hospital	12-months before	206	1.5	135.9	118.8	156.1
		After COVID-19	366	1.5	240.8	217.7	267.2
**Opioids**	Community	12-months before	5354	58.8	91.0	88.6	93.5
		After COVID-19	5331	59.1	90.1	87.8	92.6

	Hospital	12-months before	286	1.4	199.3	177.8	224.2
		After COVID-19	534	1.4	381.6	350.8	415.9
**Strong opioids**	Community	12-months before	1071	64.0	16.7	15.8	17.8
		After COVID-19	1260	64.1	19.7	18.6	20.8
	Hospital	12-months before	101	1.8	56.3	46.5	68.9
		After COVID-19	287	1.8	163.0	145.4	183.3
**Weak opioids**	Community	12-months before	5012	59.8	83.8	81.5	86.2
		After COVID-19	4896	60.1	81.5	79.3	83.8
	Hospital	12-months before	271	1.5	177.0	157.4	199.8
		After COVID-19	420	1.5	278.4	253.2	306.8
**Neuropathic pain medication**	Community	12-months before	2496	62.1	40.2	38.7	41.8
		After COVID-19	2869	62.0	46.3	44.7	48.0
	Hospital	12-months before	149	1.6	90.5	77.3	106.6
		After COVID-19	225	1.6	137.7	121.0	157.3

Table S18: Hazard ratios (95% CI) for differences in outcome event rates between patients with COVID-19 and the same patients 12 months before COVID-19 diagnosis not excluding patients with previous events.

Type of outcome	Outcome	Unadjusted HR	Lower 95% CI	Upper 95% CI	P value	Adjusted* HR	Lower 95% CI	Upper 95% CI	P value
**Symptoms**	General pain	1.23	1.09	1.39	0.0007	1.22	1.06	1.40	0.0042
	Chest pain	1.28	1.21	1.35	<0.0001	1.21	1.13	1.29	<0.0001
	Abdominal pain	1.09	1.05	1.14	0.0001	1.05	1.00	1.10	0.0439
	Headache	1.10	1.05	1.15	0.0001	1.05	1.00	1.11	0.0615
	Joint pain	0.95	0.93	0.98	0.0001	0.93	0.90	0.96	<0.0001
	Muscle pain	1.77	1.57	2.01	<0.0001	1.76	1.52	2.04	<0.0001
	Neuropathic pain	0.86	0.65	1.14	0.2971	0.89	0.65	1.23	0.4905
	Pain (all)	0.96	0.95	0.98	<0.0001	0.94	0.92	0.96	<0.0001
	Fatigue	1.68	1.59	1.79	<0.0001	1.63	1.53	1.75	<0.0001
	Fever	0.45	0.39	0.52	<0.0001	0.44	0.37	0.52	<0.0001
	Breathlessness	1.54	1.46	1.62	<0.0001	1.37	1.29	1.45	<0.0001
	Cough	0.50	0.48	0.52	<0.0001	0.48	0.46	0.50	<0.0001
	Chest tightness	1.18	0.97	1.43	0.098	1.17	0.93	1.47	0.179
	Palpitations	1.53	1.40	1.68	<0.0001	1.40	1.25	1.56	<0.0001
	Diarrhoea	0.84	0.75	0.93	0.0007	0.80	0.71	0.91	0.0004
	Nausea	1.42	1.23	1.65	<0.0001	1.24	1.05	1.47	0.0114
	Anorexia	1.00	0.83	1.20	0.9597	0.97	0.78	1.19	0.7381
	Cognitive impairment	1.14	0.92	1.40	0.2319	1.12	0.88	1.42	0.3557
	Delirium	1.19	0.91	1.55	0.1935	1.18	0.88	1.59	0.262
	Insomnia	1.55	1.40	1.71	<0.0001	1.49	1.32	1.67	<0.0001
	Dizziness	1.03	0.96	1.11	0.3656	1.02	0.94	1.10	0.6574
	Paraesthesia	1.32	1.18	1.48	<0.0001	1.33	1.17	1.51	<0.0001
	Earache	0.99	0.90	1.08	0.7648	1.02	0.92	1.13	0.7435
	Sore throat	0.45	0.43	0.48	<0.0001	0.45	0.42	0.49	<0.0001
	Smell or taste loss	5.38	3.99	7.25	<0.0001	5.40	3.74	7.81	<0.0001
	Tinnitus	1.46	1.26	1.70	<0.0001	1.34	1.13	1.60	0.0008
	Skin rash	0.84	0.79	0.88	<0.0001	0.84	0.79	0.89	<0.0001

**Diseases**	Asthma	0.83	0.81	0.86	<0.0001	0.79	0.77	0.82	<0.0001
	Lung fibrosis	2.06	1.36	3.13	0.0006	1.95	1.22	3.12	0.0055
	Heart failure	1.01	0.88	1.15	0.9357	0.93	0.79	1.09	0.3506
	IHD	0.86	0.78	0.94	0.0013	0.86	0.77	0.96	0.0084
	Hypertension	0.68	0.66	0.71	<0.0001	0.70	0.67	0.73	<0.0001
	Stroke	0.73	0.64	0.82	<0.0001	0.79	0.68	0.91	0.0013
	PAD	0.55	0.39	0.77	0.0005	0.57	0.39	0.83	0.0037
	Anaemia	1.08	1.01	1.17	0.029	1.09	1.00	1.19	0.04
	VTE	2.81	2.51	3.15	<0.0001	2.62	2.30	3.00	<0.0001
	Renal failure	1.24	1.13	1.36	<0.0001	1.22	1.10	1.35	0.0003
	GORD	1.06	0.99	1.13	0.0909	1.03	0.95	1.11	0.4792
	Liver disease	1.09	0.99	1.19	0.0658	1.08	0.97	1.20	0.1414
	Diabetes	0.84	0.82	0.86	<0.0001	0.83	0.81	0.86	<0.0001
	Adrenal disease	1.20	0.74	1.94	0.4567	1.06	0.56	2.00	0.8579
	Arthritis	0.72	0.68	0.76	<0.0001	0.80	0.74	0.85	<0.0001
	Anxiety	1.03	1.00	1.05	0.059	1.01	0.98	1.05	0.4705
	Depression	1.00	0.97	1.02	0.7427	0.97	0.93	1.01	0.0895
**Medications**	Diuretics	0.98	0.96	0.99	0.013	0.89	0.86	0.92	<0.0001
	Bronchodilators	0.91	0.89	0.92	<0.0001	0.83	0.82	0.85	<0.0001
	ICS	1.00	0.99	1.02	0.5298	0.91	0.89	0.92	<0.0001
	Paracetamol	1.06	1.04	1.09	<0.0001	1.02	0.98	1.05	0.369
	NSAIDs	1.03	1.01	1.05	0.0002	1.08	1.06	1.11	<0.0001
	Opiates (all)	1.02	1.01	1.04	0.0005	1.05	1.03	1.08	<0.0001
	Weak opiates	1.01	0.99	1.02	0.455	1.04	1.01	1.06	0.0035
	Strong opiates	1.10	1.07	1.12	<0.0001	1.12	1.08	1.17	<0.0001
	Neuropathic pain medication	1.12	1.10	1.13	<0.0001	1.11	1.08	1.14	<0.0001

Bonferroni significance threshold for symptoms=0.002, for diseases=0.003, for medications=0.005, and for HCU=0.01. *adjusted for age, sex, smoking status, BMI, and CCI with the exception of nausea, VTE, liver disease, stroke, tinnitus, and cognitive impairment which were adjusted for age, sex, smoking status and CCI due to model convergence.

Table S19: IRR (95% CI) for differences in outcome event rates between symptomatic, vaccinated patients who were not admitted to hospital for Community COVID-19 one month prior to vaccination and the same patients after vaccination.

Type of outcome	Outcome	Unadjusted IRR	Lower 95% CI	Upper 95% CI	P value	Adjusted* IRR	Lower 95% CI	Upper 95% CI	P value
**Symptoms**	General pain	0.32	0.24	0.41	<0.0001	0.43	0.29	0.62	<0.0001
	Chest pain	0.17	0.14	0.19	<0.0001	0.31	0.25	0.38	<0.0001
	Abdominal pain	0.23	0.20	0.26	<0.0001	0.34	0.29	0.41	<0.0001
	Headache	0.29	0.26	0.34	<0.0001	0.46	0.37	0.56	<0.0001
	Joint pain	0.23	0.22	0.25	<0.0001	0.36	0.33	0.39	<0.0001
	Muscle pain	0.33	0.26	0.42	<0.0001	0.46	0.33	0.64	<0.0001
	Neuropathic pain	0.27	0.14	0.54	0.0002	0.43	0.18	1.00	0.0497
	Pain (all)	0.24	0.23	0.25	<0.0001	0.38	0.35	0.40	<0.0001
	Fatigue	0.19	0.16	0.22	<0.0001	0.29	0.23	0.36	<0.0001
	Fever	0.19	0.11	0.33	<0.0001	0.50	0.29	0.87	0.0142
	Breathlessness	0.19	0.17	0.21	<0.0001	0.38	0.32	0.45	<0.0001
	Cough	0.16	0.14	0.18	<0.0001	0.33	0.27	0.4	<0.0001
	Chest tightness	0.10	0.04	0.22	<0.0001	0.14	0.06	0.34	<0.0001
	Palpitations	0.24	0.19	0.30	<0.0001	0.54	0.38	0.75	0.0004
	Diarrhoea	0.20	0.14	0.27	<0.0001	0.49	0.32	0.77	0.0018
	Nausea	0.25	0.17	0.37	<0.0001	0.33	0.19	0.56	<0.0001
	Anorexia	0.15	0.07	0.30	<0.0001	0.20	0.09	0.45	0.0001
	Cognitive impairment	0.21	0.15	0.31	<0.0001	0.32	0.18	0.58	<0.0001
	Delirium	0.21	0.13	0.36	<0.0001	0.46	0.22	0.93	0.0301
	Insomnia	0.17	0.12	0.23	<0.0001	0.29	0.19	0.45	<0.0001
	Dizziness	0.22	0.18	0.26	<0.0001	0.32	0.25	0.42	<0.0001
	Paraesthesia	0.21	0.16	0.29	<0.0001	0.42	0.29	0.60	<0.0001
	Earache	0.26	0.20	0.33	<0.0001	0.55	0.37	0.82	0.0035
	Sore throat	0.23	0.18	0.29	<0.0001	0.41	0.29	0.58	<0.0001
	Smell or taste loss	0.11	0.06	0.19	<0.0001	0.23	0.12	0.44	<0.0001

	Tinnitus	0.24	0.17	0.35	<0.0001	0.34	0.23	0.50	<0.0001
	Skin rash	0.21	0.18	0.25	<0.0001	0.30	0.23	0.40	<0.0001
**Diseases**	Asthma	0.42	0.36	0.49	<0.0001	0.55	0.40	0.77	0.0004
	Lung fibrosis	0.87	0.47	1.64	0.6752	0.34	0.17	0.66	0.0016
	Heart failure	0.91	0.62	1.34	0.6413	1.20	0.51	2.80	0.6762
	IHD	0.37	0.28	0.48	<0.0001	0.28	0.17	0.46	<0.0001
	Hypertension	0.77	0.66	0.90	0.0012	0.89	0.70	1.12	0.3283
	Stroke	0.86	0.53	1.40	0.5435	0.98	0.49	1.94	0.950
	PAD	2.46	1.20	5.07	0.0142	1.39	0.17	11.2	0.756
	Anaemia	0.58	0.44	0.76	0.0001	0.80	0.54	1.19	0.2662
	VTE	0.46	0.31	0.67	0.0001	1.11	0.68	1.80	0.6884
	Renal failure	0.67	0.48	0.93	0.0155	0.44	0.26	0.76	0.003
	GORD	0.60	0.45	0.80	0.0004	0.61	0.43	0.86	0.0051
	Liver disease	0.65	0.48	0.88	0.005	0.82	0.56	1.21	0.325
	Diabetes	0.85	0.75	0.97	0.0125	1.00	0.82	1.23	0.9905
	Adrenal disease	0.42	0.04	4.30	0.4659	0.49	0.02	10.67	0.6483
	Arthritis	0.88	0.72	1.08	0.216	0.77	0.55	1.08	0.127
	Anxiety	0.78	0.68	0.89	0.0002	0.81	0.65	1.00	0.0551
	Depression	0.94	0.81	1.08	0.3777	0.88	0.70	1.11	0.2883
**Medications**	Bronchodilators	0.79	0.75	0.82	<0.0001	0.69	0.63	0.76	<0.0001
	ICS	0.83	0.79	0.86	<0.0001	0.73	0.67	0.79	<0.0001
	Paracetamol	0.89	0.83	0.94	0.0001	0.80	0.68	0.93	0.0044
	NSAIDs	0.70	0.67	0.73	<0.0001	0.70	0.63	0.77	<0.0001
	Opiates (all)	0.74	0.71	0.76	<0.0001	0.68	0.63	0.74	<0.0001
	Weak opiates	0.69	0.65	0.72	<0.0001	0.63	0.57	0.69	<0.0001
	Strong opiates	0.82	0.78	0.87	<0.0001	0.78	0.67	0.90	0.0005
	Neuropathic pain medication	0.81	0.78	0.84	<0.0001	0.81	0.73	0.89	<0.0001
**HCU**	All HCU	0.31	0.30	0.32	<0.0001	0.36	0.34	0.37	<0.0001
	Primary care HCU	0.31	0.30	0.31	<0.0001	0.36	0.34	0.37	<0.0001
	A&E HCU	0.37	0.31	0.43	<0.0001	0.53	0.42	0.66	<0.0001

	Hospital HCU	0.25	0.20	0.33	<0.0001	0.25	0.17	0.35	<0.0001
	Outpatient HCU	0.40	0.38	0.43	<0.0001	0.38	0.35	0.42	<0.0001

Bonferroni significance threshold for symptoms=0.002, for diseases=0.003, for medications=0.005, and for HCU=0.01. *adjusted for age, sex, smoking status, BMI, and CCI with the exception of nausea, VTE, liver disease, stroke, tinnitus, and cognitive impairment which were adjusted for age, sex, smoking status and CCI due to model convergence. Adjusted IRR for diuretics did not converge.

Figure S1: Inclusion and exclusion criteria for each cohort: patients not admitted to hospital for Community COVID-19, patients admitted to hospital for COVID-19, patients included in the vaccination cohort, Community, patients included in the negative control cohort, and patients included in the influenza cohort CommunityCommunity

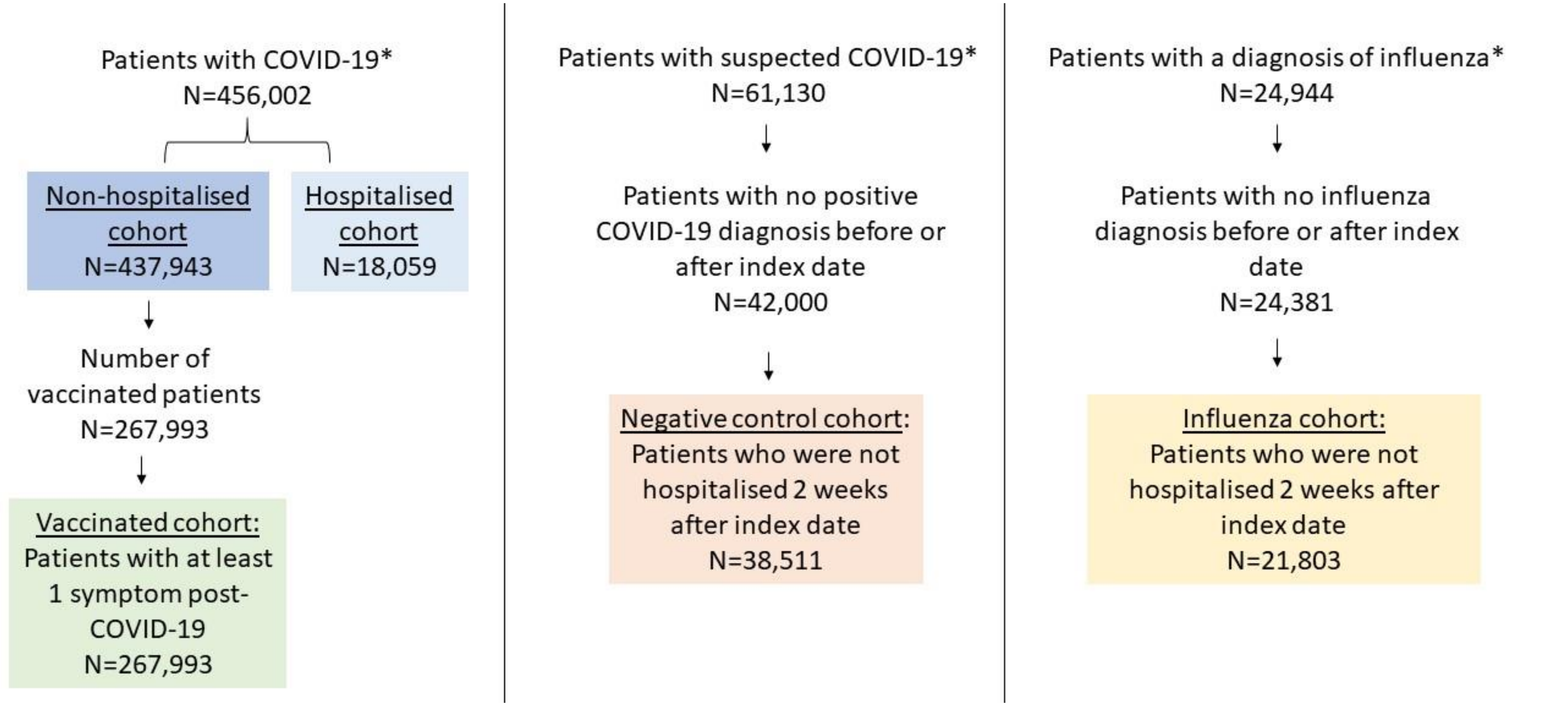

Legend: *patients over the age of 18 years old, with at least 1 day of follow-up.
